# Peculiar Expression of CD3-Epsilon in Kidney of Ginbuna Crucian Carp

**DOI:** 10.3389/fimmu.2018.01321

**Published:** 2018-06-13

**Authors:** Ryuichiro Miyazawa, Norifumi Murata, Yuta Matsuura, Yasuhiro Shibasaki, Takeshi Yabu, Teruyuki Nakanishi

**Affiliations:** ^1^Department of Veterinary Medicine, Nihon University, Fujisawa, Japan; ^2^Research Center for Fish Diseases, National Research Institute of Aquaculture, Minami-ise, Japan; ^3^Department of Pathobiology, School of Veterinary Medicine, University of Pennsylvania, Philadelphia, PA, United States; ^4^Department of Applied Biological Science, Nihon University, Fujisawa, Japan

**Keywords:** CD3-epsillon, CD4, CD8, kidney, teleost, ginbuna crucian carp, T lymphocytes

## Abstract

TCR/CD3 complex is composed of the disulfide-linked TCR-αβ heterodimer that recognizes the antigen as a peptide presented by the MHC, and non-covalently paired CD3γε- and δε-chains together with disulfide-linked ζ-chain homodimers. The CD3 chains play key roles in T cell development and T cell activation. In the present study, we found nor or extremely lower expression of CD3ε in head- and trunk-kidney lymphocytes by flow cytometric analysis, while CD3ε was expressed at the normal level in lymphocytes from thymus, spleen, intestine, gill, and peripheral blood. Furthermore, CD4-1^+^ and CD8α^+^ T cells from kidney express Zap-70, but not CD3ε, while the T cells from other tissues express both Zap-70 and CD3ε, although expression of CD3ε was low. Quantitative analysis of mRNA expression revealed that the expression level of T cell-related genes including *tcrb, cd3*ε, *zap-70*, and *lck* in CD4-1^+^ and CD8α^+^ T cells was not different between kidney and spleen. Western blot analysis showed that CD3ε band was detected in the cell lysates of spleen but not kidney. To be interested, CD3ε-positive cells greatly increased after 24 h in *in vitro* culture of kidney leukocytes. Furthermore, expression of CD3ε in both transferred kidney and spleen leukocytes was not detected or very low in kidney, while both leukocytes expressed CD3ε at normal level in spleen when kidney and spleen leukocytes were injected into the isogeneic recipient. Lower expression of CD3ε was also found in kidney T lymphocytes of goldfish and carp. These results indicate that kidney lymphocytes express no or lower level of CD3ε protein in the kidney, although the mRNA of the gene was expressed. Here, we discuss this phenomenon from the point of function of kidney as reservoir for T lymphocytes in teleost, which lacks lymph node and bone marrow.

## Introduction

The antigen receptor complex on T-cell (TCR/CD3) consists of the disulfide-linked TCR-αβ heterodimer that recognizes the antigen as a peptide presented by the MHC, and non-covalently paired CD3γε- and δε-chains together with disulfide-linked ζ-chain homodimers. In mice and humans, CD3ε and ζ chains as well as TCRα and β chains are essential for surface expression of TCR/CD3 complex, while CD3δ and γ chains are individually dispensable (1). Dynamic change of TCR/CD3 cell surface expression has been reported in resting and antigen-activated T cells (2). CD3 chains play critical roles in the various phases of thymocyte development. For instance, the removal of CD3ε led to a complete impairment of thymocyte development at the DN stage and CD3γε is the most critical for DN-to-DP transition (3). The role of CD3 chains in mature T cell activation has been also reported (4, 5). Ahmadi et al. (6) showed that co-transfer of CD3 and TCR genes into primary murine T cells enhanced TCR expression and antigen-specific T-cell function *in vitro*. Further, they demonstrated that addition of CD3 protein is effective to enhance the avidity, antitumor activity, and functional memory formation of TCR gene-modified T cells *in vivo*. In contrast, downregulation in the expression of CD3 chains in T cells and impaired immune responses have been reported in patients with malignant and/or inflammatory autoimmune diseases (7–9). These reports indicate that the critical roles of CD3 chains in mature T cell activation as well as in T cell development and signaling.

Fish CD3 genes have been reported from chondrostean fish (sterlet) and teleost [e.g., Japanese flounder, fugu, Atlantic halibut, sea bass, Atlantic salmon, and common carp (10)]. CD3 transcripts were widely expressed in teleost tissues including lymphoid tissues (e.g., thymus, head- and trunk-kidney, and spleen), mucosal tissues (e.g., gill, skin, and intestine), and peripheral blood leukocytes (PBL) (10–14). Non-mammalian vertebrates including birds, amphibians, and teleost fish possess only three types of CD3, e.g., CD3-γδ, CD3-ε, and the -ζ chain with CD3-γδ reflecting the common ancestor of mammalian CD3-γ and CD3-δ (15). The lack of interaction between chicken TCRαβ heterodimers and the human CD3 complex has been reported (16). Furthermore, there exist significant differences in the relative functions of the various CD3 chains even between mice and humans (17). In fish, however, function of CD3 chains, particularly, the role in the expression of TCR/CD3 complex remains unknown.

The obvious differences between fish and mammals are that fish lack a bone marrow and lymph nodes, and instead, the kidney is a major lymphoid organ in the teleost in addition to the thymus, spleen, and mucosa-associated lymphoid tissues (18). Teleost kidney is considered to be homologous organ to the bone marrow and lymph node in mammals (19). It has been reported that the presence of hematopoietic stem cells in the kidney of ginbuna and zebrafish (20, 21). Abundant presence of immature leukocytes or blast cells in the kidney of teleost also suggests that teleost kidney is equivalent to the bone marrow of mammals. It is well known in mammals that interactions between T cells and dendritic cells in the lymph nodes are crucial for initiating cell-mediated adaptive immune responses (22, 23) and the germinal centers are main sites for T cell-dependent immune responses (24). In fish, however, information on the tissues or sites equivalent to lymph nodes and germinal centers is limited.

In the present study, we found that CD4-1^+^ and CD8α^+^ T lymphocytes from head-and trunk-kidney express Zap-70, but not CD3ε, while T lymphocytes from tissues except kidney express both CD3ε and Zap-70. Interestingly enough, T lymphocytes from the head- and trunk-kidney become positive for the expression of CD3ε after 24 h in *in vitro* culture. Furthermore, expression of CD3ε in kidney leukocytes became positive when kidney leukocytes were injected into the isogeneic recipient and migrated into spleen. These results indicate that expression of CD3ε molecule was suppressed in the kidney. Here, we discuss this phenomenon from the point of function of kidney as reservoir for T lymphocytes in teleost, which lacks lymph node and bone marrow.

## Materials and Methods

### Experimental Fish

Triploid female ginbuna crucian carp (*Carassius auratus langsdorfii*) from Okushiri Island in Hokkaido (OB1 clone) weighing 20–30 g were used for the experiment. Offspring of OB1 clone were reproduced by naturally occurring gynogenesis artificially inseminated with loach sperm and were maintained in tanks with running water at 25 ± 1°C and fed twice daily with commercial pellets throughout the experiments.

### Identification and Characterization of Ginbuna CD3ε

To obtain the partial ginbuna CD3ε nucleotide sequences, we performed RT–PCR using primers (Table 1) designed using nucleotide sequence of zebrafish *cd3e* (NM_001326401) and Japanese flounder *cd3e* (AB081751). PCR was carried out in 40-µl reaction mixtures containing Prime STAR HS, with reaction conditions consisting of denaturation at 96°C for 2 min and 30 cycles of denaturation at 94°C for 10 s, annealing at 60°C for 10 s, polymerization at 72°C for 30 s, and extension at 72°C for 2 min. The PCR products were subcloned into the pGEM-T Easy plasmid vector by using a TA-Cloning method (Promega, Madison, WI, USA). After confirming through sequencing, 5′-RACE and 3′-RACE protocols (TaKaRa Bio, Shiga, Japan) were used to obtain full-length gCD3ε sequences using the primers in Table 1. Nucleotide and amino acid sequence were analyzed using GENETYX-WIN version 9.0 and CLUSTALW. Similarity searches were performed using BLAST against the NCBI non-redundant protein database and the Protein Data Bank. Ig domains, CXXC motif, and immunoreceptor tyrosine-based activation motifs (ITAM) were predicted using Simple Modular Architecture Research Tool. Phylogenetic analysis was performed using molecular evolutionary genetics analysis.

**Table 1:** Oligonucleotide primers used in this study.

Primer name	Sequence(5′ → 3′)		GenBank accession number
**cDNA cloning**			
*gcd3e*F1	GAAGCCGGAAAAGATGTCAG	3′RACE	
*gcd3e*F2	CAGTGAAACCGAAGAACAGC	3′RACE	
*gcd3e*R1	GGTTTGGGGGACGAGGAG	5′RACE	
*gcd3e*R2	TCTCATAGTCCGGGTTTGG	5′RACE	
*gcd3e*R3	GAGTCTGAGTGTTCAGTTTCTCATA	5′RACE	
*gcd3e*-CDS F	TGTCTTCAGGACGATACAGAACC	CDS	
*gcd3e*-CDS R	CCCTATGAATCACCAGAGTTTGA	CDS	
*gcd3e*-del-TM-F	TGTTCTGCCAGAAACAGTGACAGCAAACC	TM deletion	
*gcd3e*-del-TM-R	TCCGCTCAACTCATAGCAGTTCTCACAC	TM deletion	

**Eukaryotic expression**			
*gcd3e-*rec F	CATATGATCTGCACTGGAGGAGACAATAGGGTAG	Recombinant protein	
*gcd3e-*rec R	CATATGCTACTTATTGAGGCCTGCGTACAACCCATC	Recombinant protein	

**Expression analysis**			
*gef1*αF	ACCCCAAGGCTCTCAATCT	qPCR	AB491676
*gef1*αR	TCAACGCTCTTGATGACACC	qPCR	
*gcd3e*F	CTGCTATGAGTTGAGCGGAGTGAT	qPCR	LC378416
*gcd3e*R	CTTCGGTTTGCTGTCACTGTTTCT	qPCR	
*gtcrb*F	CCTGAAGCCCTCTGAAATCG	qPCR	AB186399
*gtcrb*R	TGCTTCCAAGGCTCCATCTT	qPCR	
*gzap70*F	TCCGAGAGAGAGAAGAATTTGGA	qPCR	
*gzap70*R	CATTGCGTATTTCCCTGATTTGT	qPCR	
*glck*F	CCATCCAGTCAAATACAGCAAA	qPCR	AB279594
*glck*R	CTTTCTCAAACCCAAGGTCATC	qPCR	

***In situ* hybridization (ISH)**			
*gcd3e* probe-t7	TAATACGACTCACTATAGGGGATGTCAGTGGAGAAGGAAA	ISH	
*gcd3e* probe-sp6	ATTTAGGTGACACTATAGAAGTCATGTATATCTCTCAGTG	ISH	

### Recombinant Protein Production

The cDNA sequences of ginbuna *cd3e* were amplified and subcloned into a pET-16b vector (Novagen, Madison, WI, USA). To increase solubility, transmembrane (TM) domain was deleted within CD3ε sequence and named as CD3e–ΔTM. The CD3e–ΔTM plasmid DNA was amplified using the primers (gCD3e-del-TM-F, gCD3e-del-TM-R in Table 1) phosphorylated with Prime STAR mutagenesis kit (TaKaRa Bio, Shiga, Japan). The construct was designated as pET–CD3e–ΔTM. The CD3e–ΔTM protein was expressed in *Escherichia coli* BL21 (DE3) pLysS cells (Novagen) that had been transformed with pET–CD3e–ΔTM. After the three chromatography purification steps, sequential His-tag affinity purification, gel filtration chromatography, and endotoxin removal, the recombinant proteins were used for immunization of rabbit.

### Production of Polyclonal Antibody

New Zealand White rabbit were immunized with the purified recombinant CD3ε according to the standard method. Rabbits were bled by cardiac puncture under deep terminal anesthesia and the serum were purified by protein G sepharose (GE Healthcare, Piscataway, NJ, USA) according to the manufacturer’s protocols and guidelines. The antibody was further purified by an affinity column, which was prepared by coupling of recombinant CD3e–ΔTM protein to NHS-activated Sepharose (GE Healthcare, Piscataway, NJ, USA) according to the manufacturer’s instructions.

### Preparation of Leukocytes

Fish were deeply anesthetized with 35 ppm ethyl-4-aminobenzoate (Benzocaine, Sigma-Aldrich, St. Louis, MO, USA), and their spinal cords were severed for euthanasia. Fish were bled from the caudal blood vessels with a heparinized syringe, and the thymus, spleen, head-kidney, trunk-kidney, gill, and intestine were dissected. To avoid contamination with blood, 10 ml of PBS with 10 U/ml of heparin (Wako Chemicals, Osaka, Japan) was injected into gill tissue through the bulbus arteriosus. All subsequent manipulations of cells were done at 4°C.

For the thymus, spleen, head-kidney, and trunk-kidney, the organs were placed on a stainless steel mesh filter (100 µm) and pressed through with 5 ml of HBSS (Nissui Pharmaceutical Co. Ltd., Tokyo, Japan) to create single-cell suspensions. For gill and intestine, the tissues were incubated with PBS containing 1 mM DTT (Wako Chemicals, Osaka, Japan) and 1 mM EDTA for 15 min after mincing with scissors. After incubation, the organs were washed and dissociated by incubating with calcium- and magnesium-free Hank’s Balanced Salt Solution (CMF-HBSS) containing 0.1 mg/ml collagenase (Wako Chemicals, Osaka, Japan), 0.1 mg/ml DNase (Sigma-Aldrich, St. Louis, MO, USA), and 5% FBS for 90 min with shaking at room temperature. Dissociated organs were disaggregated by pressing through the stainless steel mesh filter into HBSS. The buffy coat from peripheral blood and leukocytes from tissues were collected by centrifugation at 400 × *g* for 5 min at 4°C. After discarding the supernatant, 1 ml of distilled water was added to cell pellet and gently mixed with a pipette to lyse mature erythrocytes. Subsequently, 9 ml of the 0.2% FBS-HBSS was added and the cells were washed twice by centrifugation. Cell concentration and viability were determined by trypan blue dye exclusion with a hemocytometer. Viability of cells was approximately 90%.

### Cell Culture

Head- and trunk-kidney lymphocyte were suspended in RPMI1640 medium (Thermo Fisher Scientific Inc., MA, USA) supplemented with 1% ginbuna serum. The cells were seeded in 6-well plates at 1 × 10^6^ cells/2 ml/well at 25°C with 5% CO_2_ for 24 h.

### Flow Cytometry

5 × 10^6^ cells/ml of leukocytes from the various tissues were fixed with 2% paraformaldehyde (PFA) followed by cell membrane permeabilization with 0.1% saponin for 10 min. Cells were then incubated with 1:300 anti-gCD3ε antibody or Rabbi (DA1E) mAb IgG Isotype control (CST, MA, USA) for 45 min at 4°C, washed three times, and stained with 1:500 diluted Alexa Fluor^®^ 647 conjugated secondary antibody (Thermo Fisher Scientific Inc., MA, USA) against anti-gCD3ε antibody. The cells were then washed three times and served for flow cytometric analysis. Lymphocytes were gated on FS and SS dot plot and then analyzed using a FACS Canto flow cytometer (Becton Dickinson, NJ, USA).

For two-color immunofluorescence analysis of cell surface antigens along with T cell-specific intracellular markers including Zap-70 and CD3ε, kidney leukocytes were first incubated with mAbs against CD4-1(6D1, rat), CD8α (2C3, rat), IgM (B12, mouse), phagocyte (GB21, mouse), and thrombocyte (GB10, mouse) markers and then fixed with 2% PFA followed by cell membrane permeabilization with 0.1% saponin for 10 min. Cells were then incubated with 1:300 anti-gCD3ε antibody or 1:50 anti-hZap-70 (rabbit, CST, MA, USA) for 45 min at 4°C, washed three times, and stained with 1:500 diluted Alexa Fluor^®^ 488 donkey Anti-Rat IgG (H + L) antibody, Alexa Fluor^®^ 488 goat Anti-mouse IgG (H + L) antibody, and Alexa Fluor^®^ 647 goat Anti-rabbit IgG (H + L) antibody (Thermo Fisher Scientific Inc., MA, USA). A donkey anti-rat IgG antibody was used for mAbs 2C3 and 6D1, a goat anti-mouse IgG antibody was used for mAbs B12, GB21, and GB10 along with a goat anti-rabbit IgG antibody was used for mAbs gCD3ε and hZap-70. The cells were then washed three times. Lymphocytes were gated on FS and SS dot plot and lymphocytes were then analyzed for double staining with the mAbs. Doublets discrimination was performed in FSA-H/FSA-W and SSC-H/SSC-W dot plots with Flowjo 7 (TreeStar).

### Transcriptional Analysis of FACS Sorted Populations

Leukocytes from kidney and spleen were labeled with mAbs against CD4-1 and CD8α as described above. Dead cells were eliminated by 2.5 µg/ml of propidium iodide (Thermo Fisher Scientific Inc., MA, USA). Lymphocyte fraction of kidney and spleen leukocytes was gated and doublets discrimination was performed as described above. CD4-1^+^ and CD8α^+^ cells were isolated by FACS Aria II cell sorter (Becton Dickinson, NJ, USA). Purities of FACS sorted CD4-1 and CD8α were confirmed to be more than 95% when the sorted lymphocytes were re-analyzed with the mAbs used for sorting by FACS analysis. Total RNA was extracted from 5 × 10^5^ cells of FACS sorted cells using the ReliaPrep RNA Tissue Miniprep System (Promega, Madison, WI, USA) according to the manufacturer’s protocols and guidelines. cDNA was synthesized from total RNA from each sample using a High-Capacity cDNA Reverse Transcription Kit (Applied Biosystems, CA, USA) according to the manufacturer’s protocols and guidelines. mRNA expression analysis was performed by Real-time PCR using a Thermal Cycler Dice^®^ Real Time System (TaKaRa Bio, Shiga, Japan). PCR reactions were performed with 5 µl of 1:50 diluted cDNA, 12.5 µl of SYBR^®^*Premix Ex Taq* (TaKaRa Bio, Shiga, Japan), and 200 nM of each specific primer pair (Table 1) in 25 µl mixtures under the following conditions: one cycle at 95°C for 30 s, 45 cycles at 95°C for 5 s, 60°C for 30 s. Melting curve analysis showed that there was no primer dimer formation.

Target genes were amplified on the same plate with the internal control genes, *ef1a* or T cell control gene, *lck*, and the relative mRNA quantities were determined. Raw data were analyzed by the 2^−ΔΔCT^ method (25) normalized to *ef1a* or *lck*.

### Transfer of Kidney and Spleen Leukocytes Into Isogeneic Recipient

Kidney and spleen leukocytes of donor fish were prepared as mentioned above and were labeled with CFSE (Invitrogen) for detection by flow cytometry according to Toda et al. (26). Cell suspensions were adjusted to 2 × 10^6^ cells/ml and labeled with 5 µM CFSE (invitrogen) for 10 min at room temperature. The reaction was stopped by the addition of an equal volume of HBSS at 4°C followed by three washes. 100 µl of 5 × 10^7^ cells/ml of CFSE-stained kidney or spleen leukocytes were then injected into the naïve isogeneic recipient *via* caudal blood vein. Kidney and spleen cell suspension of recipient fish were prepared and the percentages of CFSE-positive cells were analyzed by FACS.

### Western Blot Analysis

Leukocytes from kidney and spleen were lysed in 25 mM Tris–HCl (pH 7.4), 150 mM NaCl, 0.1% TritonX-100, 0.05% SDS. The extracted proteins were resolved on a SDS-polyacrylamide gel and electroblotted onto a PVDF membrane as described by Yabu et al. (27). The Membrane was blocked with blocking reagent (Block-Ace, Dainippon Pharmaceutical Co., Ltd., Osaka, Japan) for 1 h at room temperature. The Membrane was probed with 1:300 Anti-gCD3ε Ab overnight at 4°C, washed five times, and incubated with horseradish peroxidase goat anti-rabbit IgG antibody (Sigma-Aldrich, St. Louis, MO, USA) diluted 1:10,000 for 1 h at room temperature, and washed an additional five times. Membranes were visualized by Western Lightning ECL Pro (Perkin Elmer, Inc., Waltham, MA, USA) and exposed to Hyperfilm ECL (GE Healthcare, Piscataway, NJ, USA).

### Immunohistological Analysis

Cryosections (8 µm) were prepared as previously reported (28). For immuno-staining, frozen sections were incubated with 1:300 diluted rabbit anti-CD3ε Ab or Rabbi (DA1E) mAbIgG Isotype control (CST, MA, USA) for 8 h at 4°C, washed, and then stained with 1:500 diluted Alexa Fluor^®^ 488 goat Anti-rabbit IgG (H + L) antibody as a secondary antibody. Sections were then washed three times and nuclei were stained with DAPI (Sigma-Aldrich, St. Louis, MO, USA). Finally, the sections were mounted with ProLong Gold anti-fade mounting medium (Life Technologies). Sections were examined by fluorescence microscopy (Olympus IX71) with a digital camera and software (Olympus DP73).

### *In situ* Hybridization (ISH)

A 1,295-bp of gCD3ε cDNA was subcloned into the pGEM-T Easy plasmid vector and then appended T7 and SP6 promoter sequence at the 5′ and 3′ terminal, respectively, by PCR reaction using primers (Table 1). The PCR product was used for sense and antisense RNA probe synthesized using DIG RNA Labeling Kit (Sigma-Aldrich, St. Louis, MO, USA). For the ISH of tissue sections, tissue samples from kidney, spleen, and thymus were fixed at 4°C for 12 h in 4% PFA. Cryosections (7 µm) were prepared on the slide. ISH was performed as described previously by Nagasawa et al. (29). The sections were then incubated in 0.0018% of BCIP (Sigma-Aldrich, St. Louis, MO, USA) and a NTMT solution containing 0.0035% of NBT (Nacalai tesque, Japan) at RT in the dark. After the color reaction had occurred, sections were washed with PBS. Finally, the sections were mounted with 70% glycerol. Sections were examined under BX51 microscope (Olympus, Tokyo, Japan).

### Statistics

Results of FCM analysis were statistically compared using two-way ANOVAs, followed by Tukey’s multiple comparisons tests to detect significant difference between means in the percentage of positive cells. A *p* value of <0.05 was considered statistically significant.

## Results

### Ginbuna CD3ε Sequence Analysis

Sequence analysis of 5′ RACE and 3′ RACE PCR product revealed that ginbuna CD3ε cDNA consists of 1,499 nucleotides with a 519 bp open reading frame encoding 173 amino acid (Figure S1A in Supplementary Material). Comparison of gCD3ε with Atlantic salmon CD3ε (NM _001123622) and human CD3ε (NM _000733.3) revealed that the polypeptide was composed of a signal peptide sequence, CXXC motif, a Ig-like domain containing two Ig-fold cysteine, and ITAM in cytoplasmic domain (Figures S1B,C in Supplementary Material). These data indicate that gCD3ε has similar feature to that of other vertebrate CD3ε. Phylogenetic analysis revealed that gCD3ε was classified into the vertebrate CD3ε group (Figure S1D in Supplementary Material).

### Specificity of Antibody Against Ginbuna CD3ε

Specificity of rabbit antibody purified with antigen column using recombinant ginbuna CD3ε (rgCD3ε) was examined. Western blot analysis showed that the antibody is specific to native ginbuna CD3ε (gCD3ε) present in thymus and spleen since positive band was detected at 20–25 kDa as expected molecular weight (Figure S2 in Supplementary Material) and disappeared after adsorption with the rgCD3ε (Figure S3 in Supplementary Material). Immuno-precipitation followed protein sequencing by LC-MS/MS revealed that the antibody recognized gCD3ε (Figure S4 in Supplementary Material).

### CD4-1^+^ and CD8α^+^ Kidney Lymphocytes Express Zap-70, but not CD3ε

**Figure 1:**
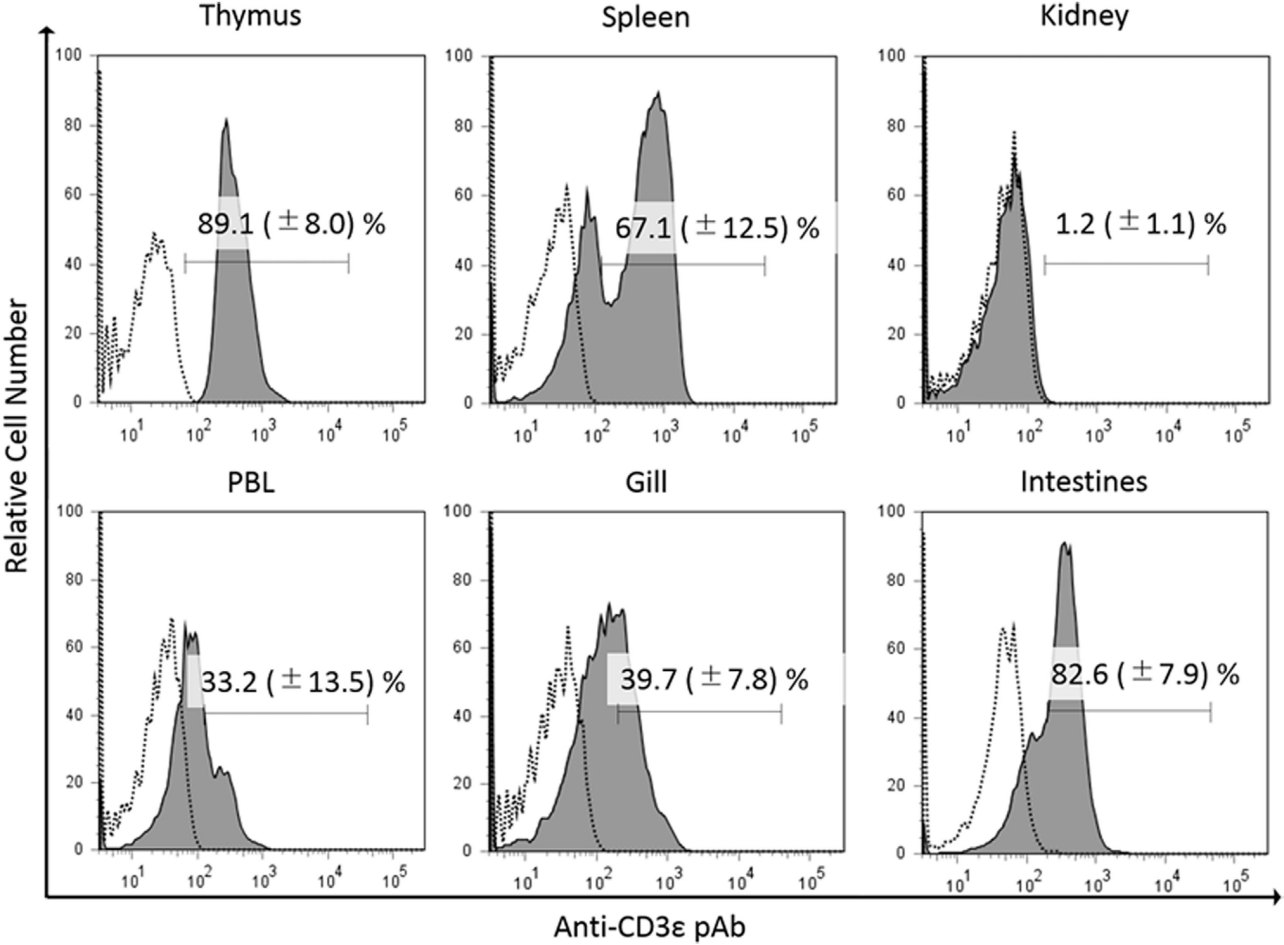
Percentages of gCD3ε-positive cells in the lymphocyte fraction of tissues. Leukocytes from thymus, spleen, trunk-kidney, gill intestine, and peripheral blood leukocytes were stained with anti-gCD3ε Ab followed by Alexa Fluor^®^ 647 goat anti-rabbit IgG. Lymphocytes were gated on FS and SS dot plot. Dotted lines show negative control stained with isotype antibody, and black solid lines with gray shadow show anti-gCD3ε Ab-positive cells. Each figure is representative of more than seven analyses with mean value and SD (mean ± SD).

**Figure 2:**
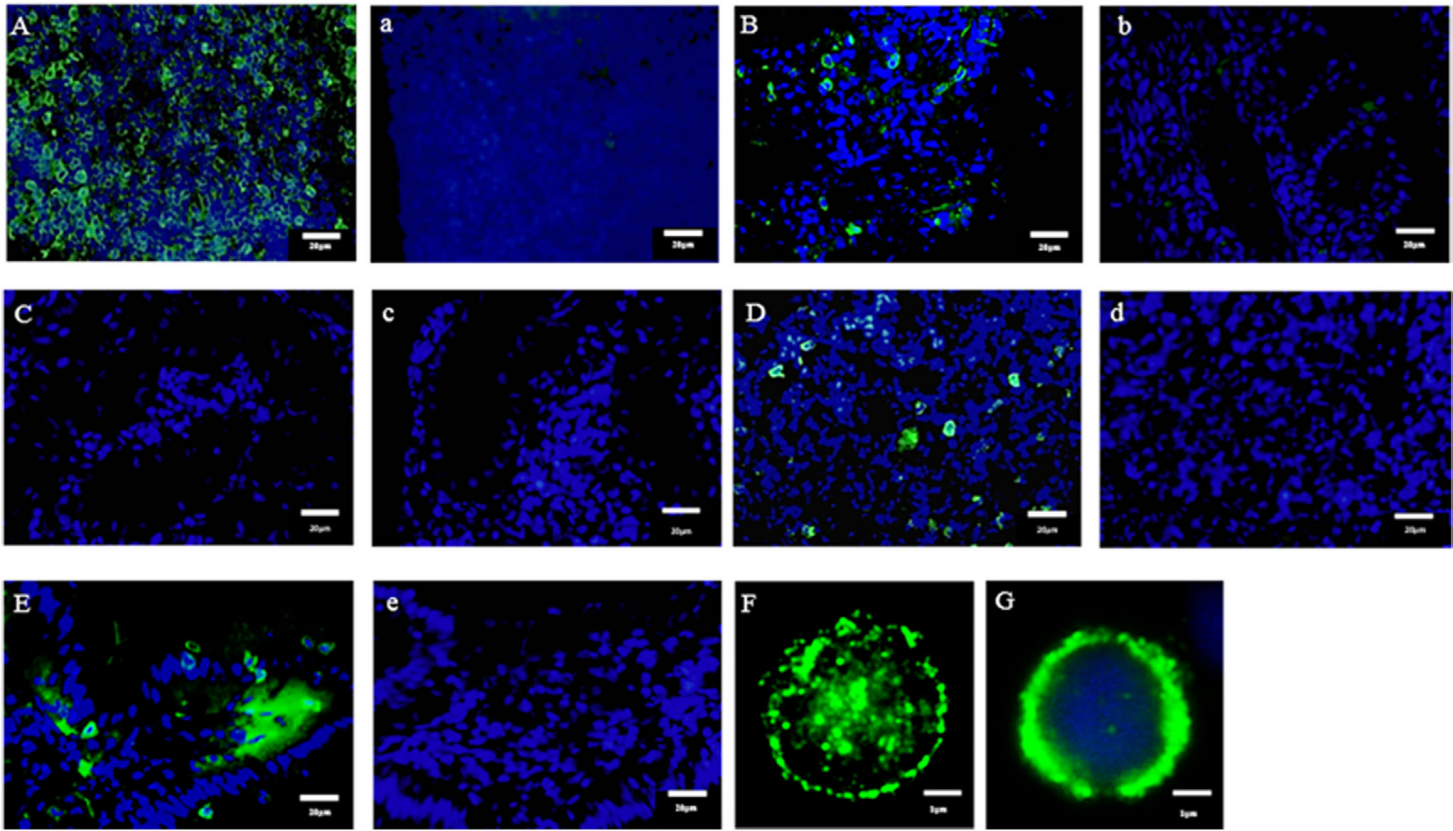
Immuno-histochemical analysis of anti-CD3ε-positive cells in tissues. Cryostat sections were stained with anti-gCD3ε Ab (A = Thymus, B = Gill, C = Trunk-Kidney, D = Spleen, and E = Intestine) or isotype Ab (a = Thymus, b = Gill, c = Trunk-Kidney, d = Spleen, and e = Intestine) followed by Alexa Fluor^®^ 488 goat anti-rabbit IgG (green). Nuclei were stained with DAPI (blue). Scale bar = 20 µm **(A–E)**. Antibody positive cells (green) were analyzed in high magnification **(F)**. Antibody positive cell (green) were merged with nuclei staining (blue) **(G)**. Scale bar = 1 µm **(F,G)**.

In the present study, we found the expression of CD3ε in the lymphocytes from thymus, spleen, intestine, gill, and peripheral blood lymphocytes but not in the lymphocytes of head- and trunk-kidney where CD4-1 and CD8α positive T cells were present (Figure 1). Immuno-histochemical analysis also showed that CD3ε-positive cells are present in all of tissues examined except kidney and the morphology of antibody-positive cells showed the typical feature of lymphocyte (Figure 2).

**Figure 3:**
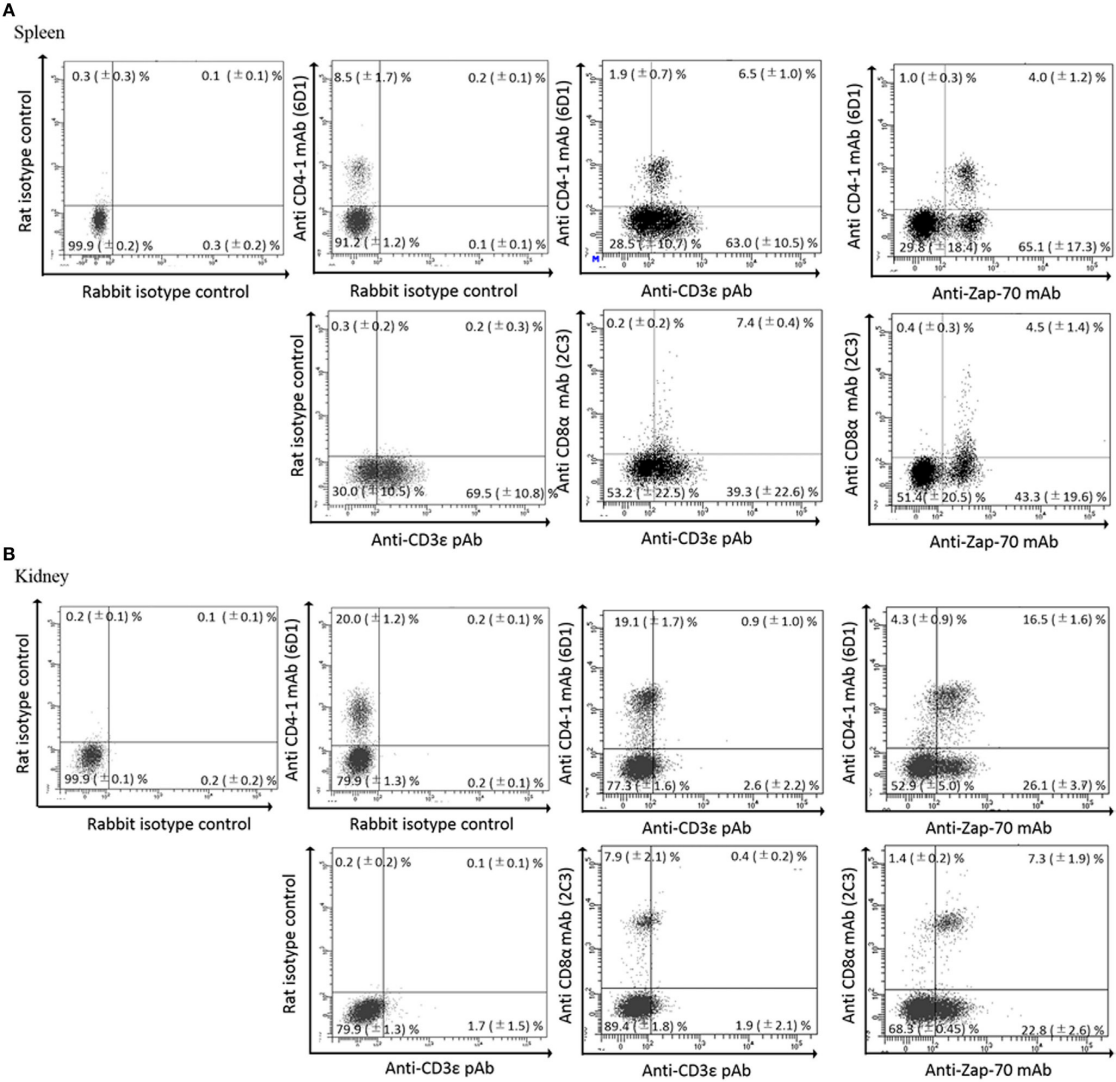

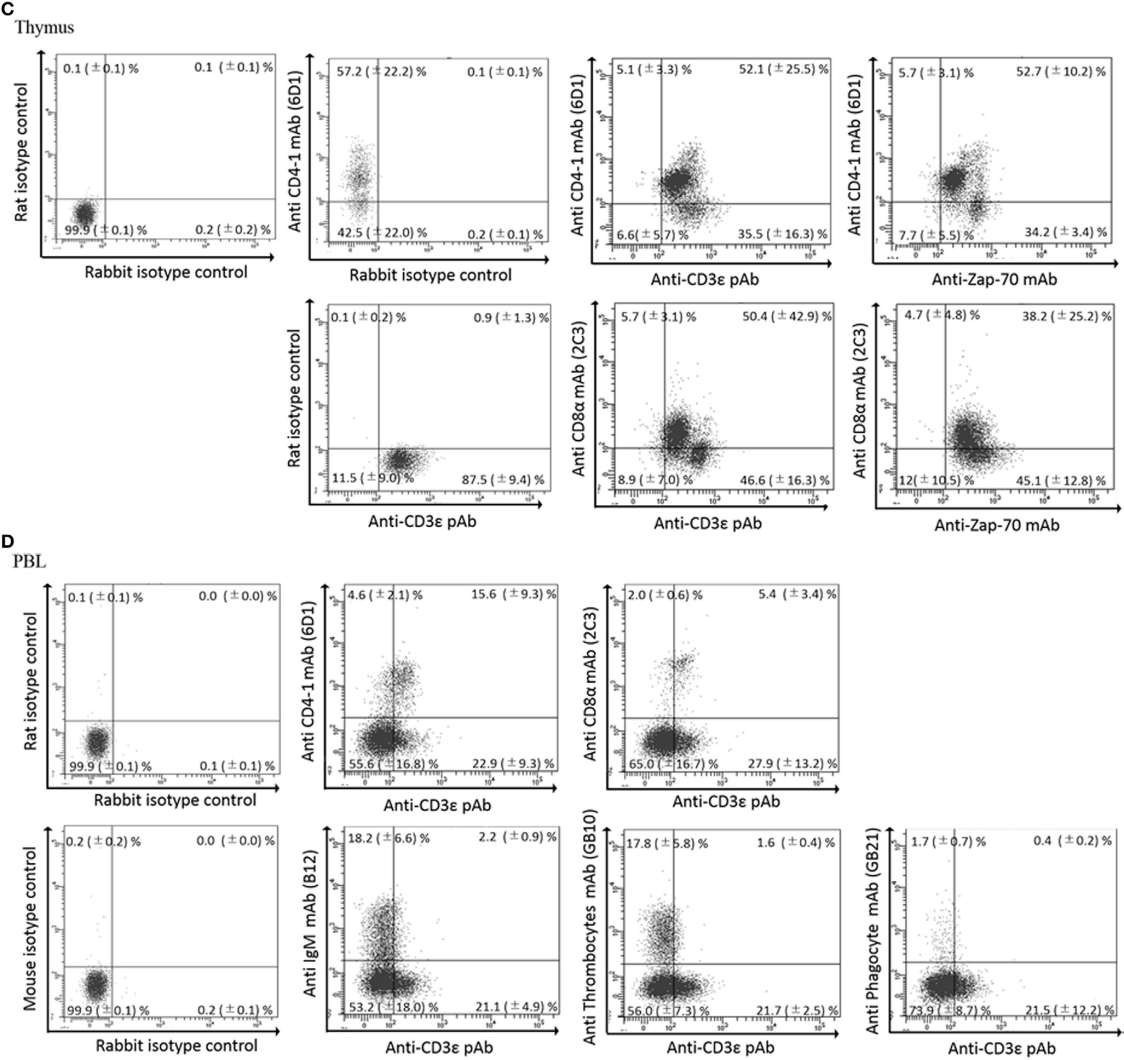
Dual fluorescence analysis of CD3ε^+^, Zap-70 with lymphocyte markers in tissues. Leukocytes from spleen, kidney, and thymus were stained with the anti-CD4-1 and CD8α mAbs followed by Alexa Fluor^®^ 488 anti-rat IgG, and stained with anti-gCD3ε Ab or anti-hZap-70 mAb followed by 647 goat anti-rabbit IgG. **(A–C)** Lymphocytes were gated on FS and SS dot plot. **(D)** Leukocytes from peripheral blood leukocytes were stained with anti-CD4-1, CD8α, IgM, phagocyte, and thrombocyte mAbs, respectively, followed by Alexa Fluor^®^ 488 goat anti-rat or mouse IgG, and stained with anti-gCD3ε Ab followed by Alexa Fluor^®^ 647 goat anti-rabbit IgG. Mean ± SD of more than three independent experiments are shown.

We previously reported the distribution of CD4-1 and CD8α positive lymphocytes in both lymphoid and non-lymphoid tissues of adult fish (30, 31). Present dual immune-fluorescence analysis revealed that CD4-1 and CD8α positive lymphocytes in the head- and trunk-kidney did not express CD3ε, while CD3ε expression was observed in other tissues, e.g., the thymus, spleen, gill, intestine, and peripheral blood (Figure 3). However, expression of Zap-70 was found in all tissues including head- and trunk-kidney. Majority of CD4-1 and CD8α positive lymphocytes express lower level of both CD3ε and Zap-70 in the thymus. Similar phenomenon was also observed in the spleen and PBL, although the tendency was more apparent in the thymus than spleen and PBL.

### FACS Sorted CD4-1^+^ and CD8α^+^ Lymphocytes Express Transcripts of T Cell-Related Markers

**Figure 4:**
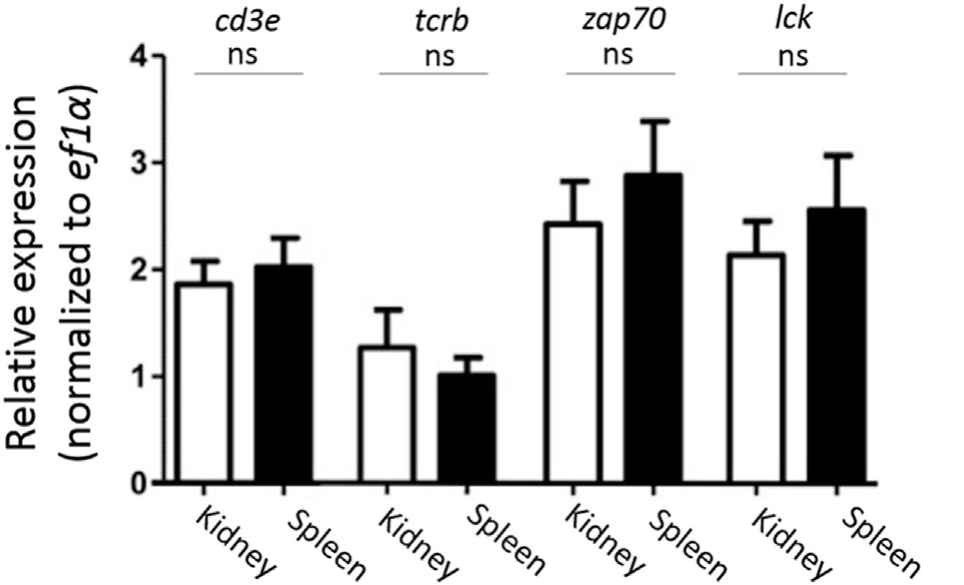
Gene expression analysis of T cell-related genes in sorted CD4-1^+^ and CD8α^+^ lymphocytes. Trunk-kidney and spleen cells were stained with anti-CD4-1 and CD8α mAbs as described in Figure 3. Lymphocytes fraction from trunk-kidney and spleen were gated on FS and SS dot plot and doublets discrimination was performed. Anti-CD4-1 and CD8α mAb positive cells were sorted by FACS. Purity of FACS sorted cells was confirmed to be more than 95%. Total RNA was prepared from 5 × 10^5^ sorted cells and used for quantitative RT-PCR analysis. mRNA expression of *cd3e, tcrb, zap-70*, and *lck* in sorted T cells was normalized to internal control gene, *ef1a*. Statistical significance was calculated using *t* tests to each gene (ns, not significant *p* > 0.05).

Since we found that CD4-1^+^ and CD8α^+^ kidney lymphocytes did not show the expression of CD3ε protein, we then examined the expression of CD3ε at mRNA level. All T cell-related genes including *tcrb, cd3*ε, *zap-70*, and *lck* were expressed to the similar extent in both kidney and spleen (Figure 4).

### Expression of CD3ε mRNA in Kidney as Well as Thymus and Spleen

**Figure 5:**
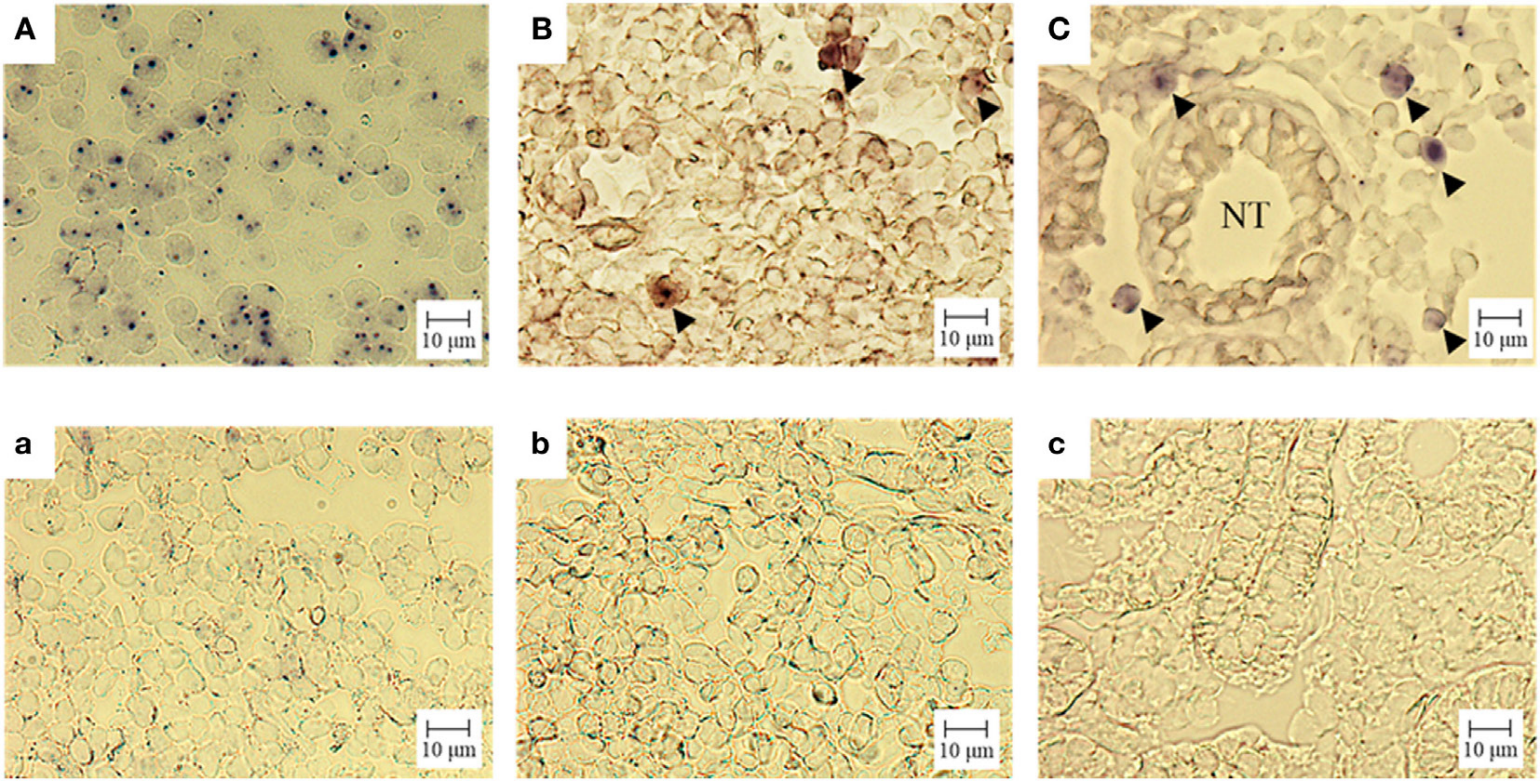
Gene expression analysis of CD3ε in tissue: *in situ* hybridization. Cryostat sections were hybridized with an antisense (A = thymus, B = spleen, C = trunk-kidney) or sense (a = thymus, b = spleen, c = trunk-kidney) probe. No unspecific staining was observed (a–c). The signal of CD3ε mRNA is observed **(A–C)**. CD3ε-expressing cells are indicated with arrow head **(B,C)**. NT represents nephric tubule in **(C)** (trunk-kidney). Scale bar = 10 µm.

We confirmed the expression of CD3ε mRNA in kidney as well as thymus and spleen by ISH (Figure 5). CD3ε mRNA positive cells were detected as small lymphocytes in the intertubular tissue of trunk-kidney (Figure 5C), although the number of positive cells in kidney was fewer when compared to thymus where most of cells were positive (Figure 5A).

### Presence of CD3ε Protein in Spleen but not Kidney Leukocytes

**Figure 6:**
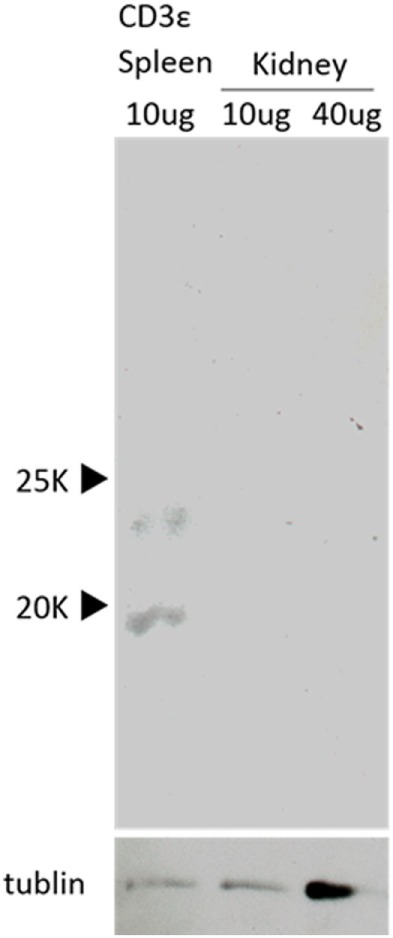
Western Blot analysis of the presence of CD3ε. Western Blot analysis with anti-CD3ε Ab of the protein samples from kidney and spleen leukocytes are shown. Both samples were normalized to tubulin (lower).

We further examined the expression of CD3ε protein in kidney and detected a clear band of 20 kDa in spleen but not in kidney by Western Blot analysis (Figure 6). No band was detected even at higher dose of samples of kidney.

### Increased Expression of CD3ε Gene in Kidney Leukocytes After *In Vitro* Culture

**Figure 7:**
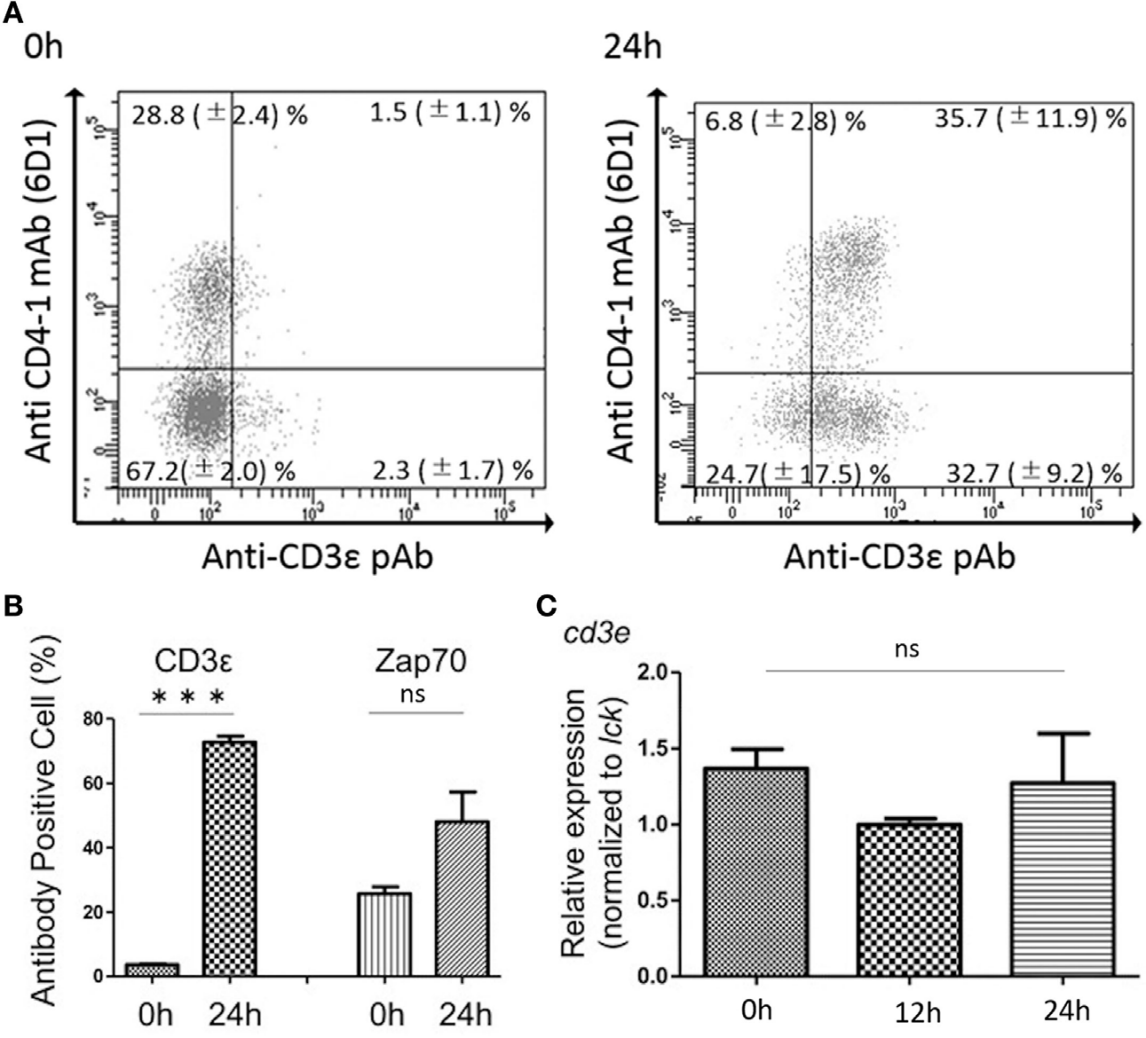
Effects of *in vitro* culture on CD3ε and Zap-70 expression in kidney lymphocytes. **(A)** Kidney leukocytes were cultured for 24 h and the expression of CD3ε was analyzed by FACS with more than 1 × 10^5^ of lymphocytes. Mean value and ±SD of more than three independent experiments are shown. **(B)** Average percentages of CD3ε and Zap-70 were calculated (*n* = 3, mean + SD). Statistical significance was calculated using *t* tests (***p < 0.005) (ns, not significant *p* > 0.05). **(C)** Total RNA was prepared from 2 × 10^6^ cultured cells and used for quantitative RT-PCR analysis. mRNA expression of *cd3e* in cultured cells was normalized to T cell control gene, *lck*. Average percentages of CD3ε and Zap-70 were calculated (*n* = 3, mean + SD). Statistical significance was calculated using one-way ANOVA (ns, not significant *p* > 0.05).

FACS analysis revealed that kidney leukocytes become positive for the expression of CD3ε after 24 h *in vitro* culture (Figure 7A). The number of CD3ε-positive cells was approximately 20 folds after the culture compared to that of leukocytes before the culture, although Zap-70-positive cells also two and three times increased (Figure 7B). However, mRNA expression of *cd3e* normalized to T cell control gene, *lck* was not different in cells before and after the culture (Figure 7C).

We also examined the changes of CD3ε expression after allo-antigen stimulation and bacterial infection. Kidney leukocytes from fish immunized with scale allografts or infected with *Edwardsiella tarda* did not show the increase of CD3ε expression (Figures S8A,B in Supplementary Material).

### Expression of CD3ε in Migrated Kidney Lymphocytes Into Recipient Spleen

**Figure 8:**
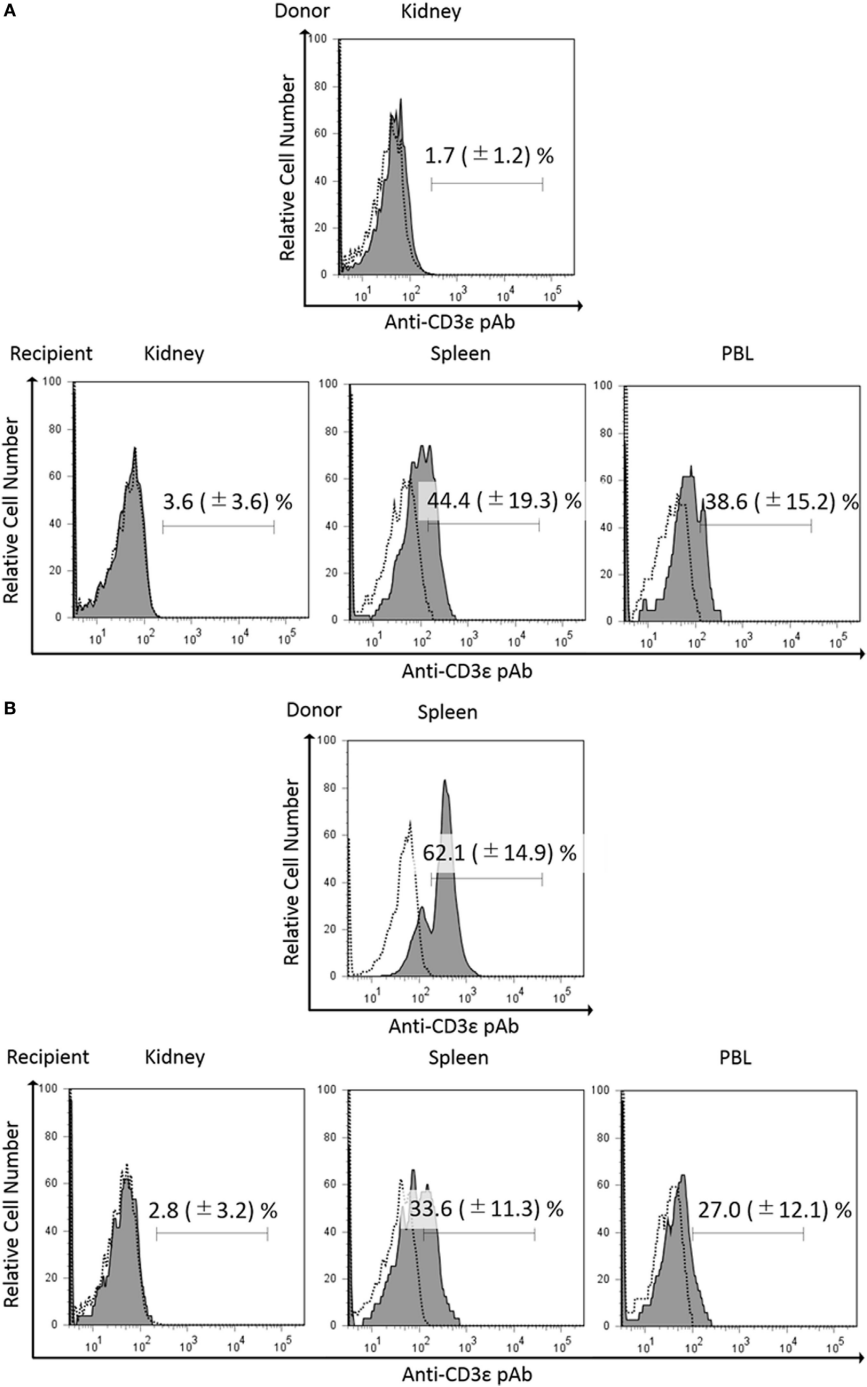
Expression of CD3ε in re-injected lymphocytes in recipient tissues. Donor leukocytes were stained with CFSE, and the expression of CD3ε was analyzed by FACS before injection. Twenty four hours after injection, lymphocytes from recipient trunk-kidney, spleen, and peripheral blood leukocytes were gated on FS and SS dot plot. Histograms show the percentages of anti-CD3ε Ab and CFSE double-positive cells derived from donors. Dotted lines show negative control stained with isotype antibody and black solid lines with gray shadow show anti-gCD3ε Ab-positive cells. Mean value and ±SD of more than three independent experiments are shown. Bars indicate gating. CFSE-labeled donor leukocytes from kidney **(A)** or spleen **(B)** were injected to the recipients.

Since we found that kidney leukocytes express CD3ε protein after *in vitro* culture, we suspect that kidney environment suppresses the expression of CD3ε protein. We then examined the CD3ε expression of kidney leukocytes migrated into other tissues of isogeneic recipient. More than 3 × 10^4^ of CFSE-positive lymphocytes were obtained and percentages of CFSE positive lymphocytes were 1.2–2.4% in kidney, spleen, and PBL of recipients fish (Figure S10 in Supplementary Material). CFSE-stained kidney leukocytes migrated into the spleen of isogeneic recipient showed the appearance of CD3ε expressing cells, while kidney cells migrated into the kidney of the recipient showed no expression (Figure 8A). Interestingly enough, CFSE-stained spleen leukocytes migrated into the kidney of the recipient failed to express CD3ε, although the leukocytes express CD3ε in the recipient spleen (Figure 8B).

## Discussion

In the present study, we found that all the leukocytes including CD4-1^+^ and CD8α^+^ T cells did not express CD3ε molecule in the head- and trunk-kidney, while lymphocytes from other tissues including thymus, spleen, intestine, gill, and peripheral blood expressed CD3ε. However, CD4-1^+^ and CD8α^+^ T cells in the kidney expressed Zap-70 as in other tissues. Furthermore, CD4-1^+^ and CD8α^+^ T cells in the head- and trunk-kidney become positive for the expression of CD3ε after 24 h in *in vitro* culture. Gene expression analysis revealed that CD4-1^+^ and CD8α^+^ T cells express both *cd3e* and *zap-70* together with *lck* and *tcrb*, and there was no difference between kidney and spleen or between before and after *in vitro* culture. To be interested, expression of CD3ε of spleen lymphocytes was suppressed in the kidney of recipient, while kidney lymphocytes expressed CD3ε in the spleen of recipient in leukocyte transfer experiment using isogeneic fish. These results suggest that the expression of CD3ε protein, but not mRNA, is suppressed in kidney environment.

Present study revealed that T cell-related genes including *tcrb, cd3*ε, *zap-70*, and *lck* were expressed in CD4-1^+^ and CD8α^+^ T cells, while FACS analysis showed that CD3ε was neither expressed in the cytoplasm nor at cell surface of lymphocytes in the kidney. Western Blot analysis also supported the results of FACS analysis. Accordingly, these results suggest that CD3ε expression is regulated at transcriptional level and CD3ε is expressed at mRNA but not protein level in ginbuna kidney. It has been reported in mammals that CD3ε and ζ chains as well as TCRα and β chains are essential for surface expression of TCR/CD3 complex (1). Taken together, it is possible that TCRα and β chains as well as CD3ε are not expressed on the cell surface or present in cytoplasm, although it is difficult to confirm due to the lack of antibody against TCR in fish including ginbuna. Critical role of CD3ε in TCR signaling has been extensively studied in mammals (3, 32). In fish, however, little is known about the role of CD3 chains in TCR signaling and transcriptional regulation of CD3 expression to date and further studies focusing on CD3ε is required.

To be interested, CD3ε-positive cells greatly increased after 24 h in *in vitro* culture of kidney leukocytes. Furthermore, kidney lymphocytes migrated into spleen of recipient expressed CD3ε as mentioned above. These results suggest that expression of CD3ε protein in lymphocytes was suppressed in the kidney. In our study on the cytotoxicity of CD8^+^ T cells against allogeneic target cells, effector kidney leukocytes are required to culture *in vitro* for at least 8 h before the mixture with the target cells to induce cytotoxic activity (unpublished data). Dynamic and rapid changes in the cell surface expression of TCR/CD3 complex have been reported and the cell-surface levels of the complex present a balance among internalization, recycling, and degradation of existing complexes (33). Furthermore, impaired cell-mediated immune responses due to decreased expression of the CD3ζ or CD3ε chain have been reported in many patients with malignant and inflammatory autoimmune diseases. For instance, downregulation of CD3ε but not CD3ζ expression in CD4^+^ and CD8^+^ T cells has been reported in patient with lung carcinomas (9). Matsuda et al. (7) have reported the decreased expression of signal-transducing CD3ζ chains in T cells from the joints and peripheral blood of rheumatoid arthritis patients. Chen et al. (8) have reported that decreased expression of both CD3ζ and CD3ε result in an increased *ex vivo* susceptibility to apoptosis of peripheral blood T cells in patients with chronic myeloid leukemia. Taken together, it is possible that the function of T cell subsets is suppressed in ginbuna kidney environment.

Zap-70 is a part of the TCR/CD3 complex and is essential for the normal development of T cells and TCR signaling. In the present study, we found that Zap-70 was expressed as protein while CD3ε was not in kidney of ginbuna by FACS analysis. In an early event in TCR activation, Zap-70 is recruited to the TCR/CD3 complex upon activation after the phosphorylation and activation of Lck and promotes recruitment and phosphorylation of downstream adaptor or scaffold proteins (34). Zap-70 is present as protein in the cytoplasm of αβ T cells and epithelial γδ T cells except for some γδ T cells in peripheral lymphoid tissues (35). In contrast, dramatic and rapid changes in the expression of CD3 chains on cell surface or in cytoplasm have been reported as mentioned above. Therefore, the difference in the expression between CD3ε and Zap-70 can be attributed to the difference in the role of two molecules.

Teleost kidney is an important hematopoietic organ (36) and has morphological similarities with the bone marrow in higher vertebrates (37). The kidney also serves as a secondary lymphoid organ involved in the induction and activation of immune responses (38). In mammals, the elimination of activated T cells at the end of immune response is essential to maintain peripheral immune tolerance and avoid excessive immune responses. Resting mature T lymphocytes in the periphery start to proliferate and then undergo the activation-induced cell death *via* apoptosis when the T cells are activated by repeated stimulation of their TCR (39). Here, we hypothesize that kidney of cyprinid fish may play a role as the reservoir of resting mature T lymphocytes. That is, T lymphocytes in kidney are suppressed in the expression of CD3ε protein and then activated after the migration into other tissues such as spleen. This hypothesis is strongly supported by our transfer experiment of CFSE-stained lymphocytes.

Flow cytometric analysis of CD3ε protein expression in tissues has been reported in several fish species. Considerably high percentages (10–40%) of CD3ε^+^ cells were detected among total head-kidney lymphocytes of rainbow trout (40–42) and Japanese flounder (43). The percentages were similar or even higher in head-kidney rather than spleen in these species, although number of CD3ε^+^ cells was relatively lower in head-kidney, spleen, and PBLs than that in thymus, gill, and intestine, and Western blot analysis showed that head-kidney preparations appeared negative or below the detection limit (40). Present results with the lack of CD3ε protein expression in kidney in cyprinid species do not agree with the abundant presence of CD3ε^+^ cells in kidney in rainbow trout and flounder. Presence of species-specific differences in fish physiology including immune responses has been reported. For instance, ultraviolet B (UVB) irradiation markedly enhanced the blood respiratory burst and cytotoxic activity in carp, although these parameters were significantly suppressed in the head kidney. In contrast, rainbow trout respiratory burst was affected only after exposure with the highest dose of UVB (44). Atlantic cod lacks the genes for CD4, MHC class II, and invariant chain involved in making and transporting MHC class II (45). However, Atlantic cod is not exceptionally susceptible to disease under natural conditions (46). Instead, Atlantic cod has a highly expanded number of MHC class I genes and unique and markedly expanded TLR genes resulting in the highest number of TLRs found in a teleost. Thus, teleost immune system is greatly diverse among species or fish groups. Accordingly, difference of CD3ε protein expression in kidney between cyprinid and other species may be attributed to the difference among species.

In the present study, we found that majority of CD4-1^+^ and CD8α^+^ lymphocytes express lower level of both CD3ε and Zap-70 in the thymus, spleen, and PBL when compared to CD4-1^−^, CD8α^−^, and CD3ε^+^ cells. We found that there are two sIgM-positive lymphocytes in the spleen and kidney of ginbuna, sIgM^low^ and sIgM^high^. In our previous study, sIgM-positive lymphocytes showed moderate non-specific cytotoxicity, while CD8α^+^ lymphocytes exhibited high-specific killing of allogeneic target cell lines when effector donor fish were sensitized by alloantigens (26). Furthermore, we also found that sIgM^low^ cells expressing granzyme and perfolin genes exhibited moderate cytotoxicity against allogeneic target cell lines, although sIgM^high^ did not express these genes and showed no cytotoxicity suggesting that sIgM^high^ cells are B lymphocytes (unpublished data). It has been reported that activated NK cells express cytoplasmic CD3ε protein in human adult and NK cell clones established from human fetal liver express CD3γ,δ,ε complexes in the cytoplasm but not cell surface (47). Similarly, Phillips et al. (48) has reported that fetal NK cells mediate cytolytic function and express cytoplasmic CD3δ,ε proteins. Furthermore, it has been reported that fish NK cells also express Fc receptor (49). Taken together with previous studies, present results suggest that sIgM^low^ cells among CD4-1^−^, CD8α^−^, and CD3ε^+^ cells are NK cells with Fc receptor.

Present study revealed that considerable numbers of CD3ε^+^CD4-1^−^CD8α^−^ cell populations (approx. 40%) were present in spleen of carp and goldfish as well as ginbuna. It is difficult to conclude that all the populations are NK cells. Recently, lymphocyte populations involved in innate immunity have been discovered in human and mice as “innate lymphocytes,” which includes NK cells and three groups of innate lymphoid cells (ILCs) (50, 51) and “innate-like lymphocytes” including γδT cells invariant NKT (iNKT) cells and mucosal-associated invariant T (MAIT) cells (52). Intracellular expression of CD3ε has been reported in CD4^+^ ILC1 (53), and it is well known that γδT cells, iNKT, and MAIT cells express CD3ε as innate T cells. Accordingly, we suspect that some of “innate lymphocytes” or “innate-like lymphocytes” are included in CD3ε^+^CD4-1^−^CD8α^−^ cells in spleen. In fish, however, no information is available on these newly discovered lymphocyte-like populations except NK cells. Further study on lymphocyte-like populations involved in innate immunity in fish is required to solve the problem.

In conclusion, CD4-1^+^ and CD8α^+^ T cells express CD3ε mRNA but not molecule in the head- and trunk-kidney of ginbuna suggesting the lack of surface expression of TCR/CD3 complex. CD4-1^+^ and CD8α^+^ T cells in the kidney become positive for the expression of CD3ε after 24 h in *in vitro* culture and kidney lymphocytes expressed CD3ε in the spleen of recipient when transferred into other individuals belonging to the same clone. These finding indicate that expression of CD3ε was suppressed in kidney and suggest that teleost kidney plays a role as the reservoir of resting mature T lymphocytes, although the precise mechanism of the suppression in CD3ε expression in fish kidney remain unknown.

## Ethics Statement

All of the experiments described comply with the Guidelines of Nihon University Rules concerning Animal Care and Use and have been approved by the Nihon University Animal Care and Use Committee (No. AP12B014).

## Author Contributions

RM and NM performed all the experimental work, with help from YM, YS, and TY. TN and RM designed the experiments and wrote the main body of the paper, with contributions from YS.

## Conflict of Interest Statement

The authors declare that the research was conducted in the absence of any commercial or financial relationships that could be construed as a potential conflict of interest.

## Supplementary Material

Figure S1 Ginbuna CD3ε sequence. **(A)** Ginbuna CD3ε sequence. Nucleotide and amino acid sequence of ginbuna CD3ε are shown. Predicted signal peptide, extracellular domain, transmembrane region, and cytoplasmic domain are labeled, CXXC motif and ITAM are boxed. Amino acid numbers are at right. **(B)** Schematic illustration of gCD3ε. Ginbuna CD3ε can be divided into Ig-like domain, CXXC motif, transmembrane region, and ITAM. **(C)** Amino acid alignment of gCD3ε with Atlantic salmon (NM _001123622) and human (NM _000733.3) CD3ε sequences. The predicted signal peptide and domains are labeled. Residues similar/identical with gCD3ε are gray/black shade respectively. Ig-fold cysteine, CXXC motif and ITAM are boxed, and gaps (–) are indicated. Amino acid numbers are at right. **(D)** Comparison of ginbuna CD3ε with vertebrates CD3ε, CD3γ, CD3δ, and CD3ζ. Accession number of CD3 sequences are carp CD3ε (XM_019126514.1), takifugu (ta)CD3ε (NM_001037982.1), taCD3γ/δ (NM_001037983.1), taCD3ζ (XM_011608167.1), Japanese flounder (ja)CD3ε (XM_020094967.1), jaCD3γ (XM_020094974.1), jaCD3ζ (XM_020112573.1), salmon (sa)CD3ε (NP_001117094.1), saCD3δ (XM_014162423.1), saCD3ζ (XM_014164569.1), chicken CD3ε (NM_206904.1), mouse (mo)CD3ε (NM_007648.4), moCD3γ (NM_009850.2), moCD3δ, moCD3ζ (NM_001113391.2), human (hu)CD3ε (NP_000724.1), huCD3γ (EF444965.1), huCD3δ (EF444964.1), and huCD3ζ (AK128376.1).

FIGURE S2 Immuno-precipitation and Western blot analysis. After immuno-precipitation of the thymus and spleen protein samples with anti-gCD3ε Ab, the proteins were detected by western blotting with anti-gCD3ε Ab. Both samples show some bands around 20–25 kDa expected to be gCD3ε and single band around 55 kDa expected to be heavy-chain.

FIGURE S3 Specificity test of rabbit serum by immune-absorption. Absorption test of gCD3ε Ab was performed using transmembrane deletion mutant (TMDM) recombinant gCD3ε protein. Western blot analysis shows no band when anti-gCD3ε Ab was absorbed with antigen (TMDM rgCD3ε, right), while the Ab not absorbed with the antigen shows positive band (left).

FIGURE S4 Protein sequencing by LC-MS/MS. Protein sequencing was determined using a protein band reactive with anti-gCD3ε Ab detected by Western blot. LC-MS/MS revealed 28 amino acid residues (gray highlight) and 16.1% of residues matched with gCD3ε amino acid sequence **(A)**. Mass spectrum and fragmentation tables of each amino acid fragments are shown in **(B,C)**, respectively. Peptide sequencing is indicated by matching b ion (red) and y ion (blue) fragments.

FIGURE S5 Expression analysis of CD3ε in ginbuna tissues by RT-PCR. Total RNA was prepared from peripheral blood leukocytes (PBL), thymus, head-kidney, trunk-kidney, spleen, liver, ovary, intestine, skin, and gill tissues, and used for RT-PCR analysis. ef-1a was used as an internal control. Numbers to the right indicate PCR cycles.

FIGURE S6 Gene expression analysis of T and B cell related genes in sorted CD3ε^+^ lymphocytes. Spleen cells were stained with anti-CD3ε Ab as described. Lymphocytes fraction from spleen were gated on FS and SS dot plot and anti-CD3ε Ab positive cells were sorted by FACS. Total RNA was prepared from 1×10^6^ sorted cells and used for RT-PCR analysis. mRNA expression of cd3e, cd4-, tcrb, lck, and igm in sorted lymphocytes were shown. ef-1a was used as an internal control. Numbers to the right indicate PCR cycles.

FIGURE S7 CD3ε expression in tissues of other cyprinid species. Spleen and kidney leukocytes from carp **(A)** and goldfish **(B)** were stained with anti-CD4-1 and CD8α mAbs followed by Alexa Fluor® 488 anti-rat IgG, and stained with anti-gCD3ε Ab or anti-hZAP-70 mAb followed by 647 goat anti-rabbit IgG. Lymphocytes were gated on FS and SS dot plot. Mean ± SD of more than three independent experiments are shown.

FIGURE S8 Modulation of CD3ε expression. After allo-antigen stimulation **(A)** or Edwardsiella tarda infection **(B)**, kidney lymphocyte was stained with anti-gCD3ε as described above and analyzed by FACS. Mean ± SD of more than three independent experiments are shown. Statistical significance was calculated using t tests to each gene (ns, not significant; p > 0.05).

FIGURE S9 FS and SS dot plots of kidney and spleen leukocytes. Lymphocytes, myeloid cells, and granulocytes were gated on FSC_low_ SSC_low_, FSC_high_ SSC_low_, and FSC_med_ SSC_high_ population, respectively **(A)**. Lymphocytes from kidney and spleen were gated on FSC_low_ SSC_low_ population. The percentages of anti-CD3ε pAb positive cells were shown in the histogram **(B)**.

FIGURE S10 Migration of donor cells in recipient organs. CFSE-labeled donor cells were detected in recipient kidney, spleen, and peripheral blood leukocytes (PBL) on the histograms. Mean ± SD of more than three independent experiments are shown.

FIGURE S11 Effect of in vitro culture on the leukocytes composition. Before in vitro (0 h) culture, leukocytes from kidney are composed of 46.2% of granulocytes, 11.1% of monocytes, and 39.3 % of lymphocytes. Similarly, after in vitro (24 h) culture, leukocytes from kidney are composed of with 50.0% of granulocytes, 7.6% of monocytes, and 32.2% of lymphocytes.

## References

[1] Kappes DJ Tonegawa S. Surface expression of alternative forms of the TCR/CD3 complex. Proc Natl Acad Sci U S A (1991) 88:10619–23. 10.1073/pnas.88.23.10619 1835791 PMC52981

[2] Liu H Rhodes M Wiest DL Vignali DA. On the dynamics of TCR:CD3 complex cell surface expression and downmodulation. Immunity (2000) 13:665–75. 10.1016/S1074-7613(00)00066-2 11114379

[3] Dave VP Hierarchial role of CD3 chains in thymocyte development. Immunol Rev (2009) 232:22–33. 10.1111/j.1600-065X.2009.00835.x 19909353

[4] Hayes SM Shores EW Love PE An architectural perspective on signaling by the pre-, αβ and γδ T cell receptors. Immunol Rev (2003) 191:28–37. 10.1034/j.1600-065X.2003.00011.x 12614349

[5] Brodeur JF Li S da Silva Martins M Larose L Dave VP. Critical and multiple roles for the CD3epsilon intracytoplasmic tail in double negative to double positive thymocyte differentiation. J Immunol (2009) 182:4844–53. 10.4049/jimmunol.0803679 19342663

[6] Ahmadi M King JW Xue SA Voisine C Holler A Wright GP CD3 limits the efficacy of TCR gene therapy *in vivo*. Blood (2011) 118:3528–37. 10.1182/blood-2011-04-346338 21750319

[7] Matsuda M Ulfgren AK Lenkei R Petersson M Ochoa AC Lindblad S Decreased expression of signal-transducing CD3 zeta chains in T cells from the joints and peripheral blood of rheumatoid arthritis patients. Scand J Immunol (1998) 47:254–62. 10.1046/j.1365-3083.1998.00296.x 9519864

[8] Chen X Woiciechowsky A Raffegerst S Schendel D Kolb HJ Roskrow M. Impaired expression of the CD3-zeta chain in peripheral blood T cells of patients with chronic myeloid leukaemia results in an increased susceptibility to apoptosis. Br J Haematol (2000) 111:817–25. 10.1046/j.1365-2141.2000.02415.x 11122143

[9] Prado-Garcia H Aguilar-Cazares D Meneses-Flores M Morales-Fuentes J Lopez-Gonzalez JS Lung carcinomas do not induce T-cell apoptosis via the Fas/Fas ligand pathway but down-regulate CD3 epsilon expression. Cancer Immunol Immunother (2008) 57:325–36. 10.1007/s00262-007-0372-6 17668204 PMC11030893

[10] Tang X Qin Y Sheng X Xing J Zhan W. Characterization of CD3^+^ T lymphocytes of Japanese flounder (*Paralichthys olivaceus*) and its response after immunization with formalin- inactivated *Edwardsiella tarda*. Fish Shellfish Immunol (2017) 63:220–7. 10.1016/j.fsi.2017.02.024 28232197

[11] Araki K Suetake H Kikuchi K Suzuki Y Characterization and expression analysis of CD3ε and CD3 g/d in fugu, *Takifugu rubripes*. Immunogenetics (2005) 57:158–63. 10.1007/s00251-005-0772-8 15756549

[12] Shang N Sun XF Hu W Wang YP Guo QL Molecular cloning and characterization of common carp (*Cyprinus carpio* L.) TCR g and CD3 g/d chains. Fish Shellfish Immunol (2008) 24:412–25. 10.1016/j.fsi.2007.12.007 18272397

[13] Øvergård AC Hordvik I Nerland AH Eikeland G Patel S. Cloning and expression analysis of Atlantic halibut (*Hippoglossus hippoglossus*) CD3 genes. Fish Shellfish Immunol (2009) 27:707–13. 10.1016/j.fsi.2009.08.011 19744563

[14] Randelli E Scapigliati G Buonocore F CD3g/d in sea bass (*Dicentrarchus labrax*): molecular characterization and expression analysis. Results Immunol (2011) 1:31–5. 10.1016/j.rinim.2011.08.003 24371550 PMC3787811

[15] Laing KJ Hansen JD. Fish T cells: recent advances through genomics. Dev Comp Immunol (2011) 35:1282–95. 10.1016/j.dci.2011.03.004 21414347

[16] Gouaillard C Huchenq-Champagne A Arnaud J Chen Cl CL Rubin B Evolution of T cell receptor (TCR) α β heterodimer assembly with the CD3 complex. Eur J Immunol (2001) 31:3798–805. 10.1002/1521-4141(200112)31:12<3798::AID-IMMU3798>3.0.CO;2-Z 11745401

[17] Dave VP Role of CD3ε-mediated signaling in T-cell development and function. Crit Rev Immunol (2011) 31:73–84. 10.1615/CritRevImmunol.v31.i1.70 21395513

[18] Press CMcL Evensen Ø The morphology of the immune system in teleost fishes. Fish Shellfish Immunol (1999) 9:309–18. 10.1006/fsim.1998.0181

[19] Zapata AG Chibá A Varas A Cells and tissues of the immune system of fish. In: Iwama G Nakanishi T, editors. The Fish Immune System: Organism, Pathogen, and Environment. San Diego: Academic Press (1996). p. 1–62.

[20] Kobayashi I Moritomo T Ototake M Nakanishi T. Isolation of side population cells from ginbuna carp (*Carassius auratus langsdorfii*) kidney hematopoietic tissues. Dev Comp Immunol (2007) 31:696–707. 10.1016/j.dci.2006.10.003 17129605

[21] Kobayashi I Saito K Moritomo T Araki K Takizawa F Nakanishi T. Characterization and localization of side population (SP) cells in zebrafish kidney hematopoietic tissue. Blood (2008) 111:1131–7. 10.1182/blood-2007-08-104299 17932252

[22] Breart B Bousso P. Cellular orchestration of T cell priming in lymph nodes. Curr Opin Immunol (2006) 18:483–90. 10.1016/j.coi.2006.05.006 16765578

[23] Hugues S. Dynamics of dendritic cell-T cell interactions: a role in T cell outcome. Semin Immunopathol (2010) 32:227–38. 10.1007/s00281-010-0211-2 20607241

[24] Ramiscal RR Vinuesa CG T-cell subsets in the germinal center. Immunol Rev (2013) 252:146–55. 10.1111/imr.12031 23405902

[25] Livak KJ Schmittgen TD Analysis of relative gene expression data using real-time quantitative PCR and the 2^−ΔΔ^*^C^*_T_ method. Methods (2001) 25:402–8. 10.1006/meth.2001.1262 11846609

[26] Toda H Shibasaski Y Koike T Ohtani M Takizawa F Ototake M Alloantigen-specific killing is mediated by CD8-positive T cells in fish. Dev Comp Immunol (2009) 33:646–52. 10.1016/j.dci.2008.11.008 19073209

[27] Yabu T Toda H Shibasaki Y Araki K Yamashita M Anzai H Antiviral protection mechanisms mediated by ginbuna crucian carp interferon gamma isoforms 1 and 2 through two distinct interferon gamma receptors. J Biochem (2011) 150:635–48. 10.1093/jb/mvr108 21903635

[28] Shibasaki Y Matsuura Y Toda H Imabayashi N Nishino T Uzumaki K Kinetics of lymphocyte subpopulations in allogeneic grafted scales of ginbuna crucian carp. Dev Comp Immunol (2015) 52:75–80. 10.1016/j.dci.2015.04.013 25917429

[29] Nagasawa K Takeuchi Y Miwa M Higuchi K Morita T Mitsuboshi T cDNA cloning and expression analysis of a *vasa*-like gene in pacific bluefin tuna *Thunnus orientalis*. Fish Sci (2009) 75:71–9. 10.1007/s12562-008-0021-9

[30] Toda H Saito Y Koike T Takizawa F Araki K Yabu T Conservation of characteristics and functions of CD4 positive lymphocytes in a teleost fish. Dev Comp Immunol (2011) 35:650–60. 10.1016/j.dci.2011.01.013 21272597

[31] Khallaf MA Ogawa S Toda H Matsuura Y Miyazawa R Nakanishi T Percentages of CD4-1^+^ and CD8α^+^ T cells in different tissues at various developmental stages in ginbuna crucian carp. Fish Pathol (2018) 53:10–8. 10.3147/jsfp.53.10

[32] Borroto A Abia D Alarcón B. Crammed signaling motifs in the T-cell receptor. Immunol Lett (2014) 161:113–7. 10.1016/j.imlet.2014.05.007 24877875

[33] Rovira-Clavé X Angulo-Ibáñez M Tournier C Reina M Espel E. Dual role of ERK5 in the regulation of T cell receptor expression at the T cell surface. J Leukoc Biol (2016) 99:143–52. 10.1189/jlb.2A0115-034R 26302753

[34] Ngoenkam J Schamel WW Pongcharoen S. Selected signalling proteins recruited to the T-cell receptor-CD3 complex. Immunology (2018) 153:42–50. 10.1111/imm.12809 28771705 PMC5721247

[35] Kadlecek TA van Oers NS Lefrancois L Olson S Finlay D Chu DH Differential requirements for ZAP-70 in TCR signaling and T cell development. J Immunol (1998) 161:4688–94. 9794398

[36] Fänge R. Lymphoid organs in sturgeons (Acipenseridae). Vet Immunol Immunopathol (1986) 12:153–61. 10.1016/0165-2427(86)90119-4 3765338

[37] Meseguer J López-Ruiz A Garcí-Ayala A. Reticulo-endothelial stroma of the head-kidney from the seawater teleost gilthead seabream (*Sparus aurata* L.): an ultrastructural and cytochemical study. Anat Rec (1995) 241:303–9. 10.1002/ar.1092410303 7755169

[38] Kaattari SL Irwin MJ. Salmonid spleen and anterior kidney harbor populations of lymphocytes with different B cell repertoires. Dev Comp Immunol (1985) 9:433–44. 10.1016/0145-305X(85)90006-0 3899761

[39] Sikora E. Activation-induced and damage-induced cell death in aging human T cells. Mech Ageing Dev (2015) 151:85–92. 10.1016/j.mad.2015.03.011 25843236

[40] Koppang EO Fischer U Moore L Tranulis MA Dijkstra JM Köllner B Salmonid T cells assemble in the thymus, spleen and in novel interbranchial lymphoid tissue. J Anat (2010) 217:728–39. 10.1111/j.1469-7580.2010.01305.x 20880086 PMC3039185

[41] Boardman T Warner C Ramirez-Gomez F Matrisciano J Bromage E. Characterization of an anti-rainbow trout (*Oncorhynchus mykiss*) CD3ε monoclonal antibody. Vet Immunol Immunopathol (2012) 145:511–5. 10.1016/j.vetimm.2011.11.017 22188783

[42] Maisey K Montero R Corripio-Miyar Y Toro-Ascuy D Valenzuela B Reyes-Cerpa S Isolation and characterization of salmonid CD4+ T cells. J Immunol (2016) 196:4150–63. 10.4049/jimmunol.1500439 27053758

[43] Xing J Ma J Tang X Sheng X Zhan W. Characterizations of CD4-1, CD4-2 and CD8β T cell subpopulations in peripheral blood leucocytes, spleen and head kidney of Japanese flounder (*Paralichthys olivaceus*). Mol Immunol (2017) 85:155–65. 10.1016/j.molimm.2017.02.015 28260650

[44] Markkula SE Salo MH Rikalainen AK Jokinen EI Different sensitivity of carp (*Cyprinus carpio*) and rainbowtrout (*Oncorhynchus mykiss*) to the immunomodulatoryeffects of UVB irradiation. Fish Shellfish Immunol (2017) 21:70–9. 10.1016/j.fsi.2005.10.007 16376572

[45] Star B Nederbragt JA Jentoft S Grimholt U Malmstrøm M Gregers FT The genome sequence of Atlantic cod reveals aunique immune system. Nature (2011) 477:207–10. 10.1038/nature10342 21832995 PMC3537168

[46] Pilstrom L Warp WG Stromberg S Why is the antibody response of Atlantic cod so poor? The search for a genetic explanation. Fish Sci (2005) 71:961–71. 10.1111/j.1444-2906.2005.01052.x

[47] Lanier LL Chang C Spits H Phillips JH. Expression of cytoplasmic CD3 epsilon proteins in activated human adult natural killer (NK) cells and CD3 gamma, delta, epsilon complexes in fetal NK cells. Implications for the relationship of NK and T lymphocytes. J Immunol (1992) 149:1876–80. 1387664

[48] Phillips JH Hori T Nagler A Bhat N Spits H Lanier LL. Ontogeny of human natural killer (NK) cells: fetal NK cells mediate cytolytic function and express cytoplasmic CD3 epsilon, delta proteins. J Exp Med (1992) 175:1055–66. 10.1084/jem.175.4.1055 1372642 PMC2119193

[49] Shen L Stuge TB Evenhuis JP Bengtén E Wilson M Chinchar VG Channel catfish NK-like cells are armed with IgM via a putative FcmicroR. Dev Comp Immunol (2003) 27:699–714. 10.1016/S0145-305X(03)00042-9 12798366

[50] Artis D Spits H. The biology of innate lymphoid cells. Nature (2015) 517:293–301. 10.1038/nature14189 25592534

[51] Eberl G Colonna M Di Santo JP McKenzie AN Innate lymphoid cells: a new paradigm in immunology. Science (2015) 348(6237):aaa6566 10.1126/science.aaa6566 25999512 PMC5658207

[52] Bennett MS Round JL Leung DT. Innate-like lymphocytes in intestinal infections. Curr Opin Infect Dis (2015) 28:457–63. 10.1097/QCO.0000000000000189 26270655 PMC4925623

[53] Roan F Stoklasek TA Whalen E Molitor JA Bluestone JA Buckner JH CD4+ group 1 innate lymphoid cells (ILC) form a functionally distinct ILC subset that is increased in systemic sclerosis. J Immunol (2016) 96:2051–62. 10.4049/jimmunol.1501491 PMC4761490 26826243

