# Supplementary material for: Peculiar Expression of CD3-Epsilon in Kidney of Ginbuna Crucian Carp
Source: Front Immunol. 2018 Jun 13;9:1321. doi: 10.3389/fimmu.2018.01321 (PMC6008321; doi:10.3389/fimmu.2018.01321)
Supplement: Figure S1 — Ginbuna CD3ε sequence. (A) Ginbuna CD3ε sequence. Nucleotide and amino acid sequence of ginbuna CD3ε are shown. Predicted signal peptide, extracellular domain, transmembrane region, and cytoplasmic domain are labeled, CXXC motif and ITAM are boxed. Amino acid numbers are at right. (B) Schematic illustration of gCD3ε. Ginbuna CD3ε can be divided into Ig-like domain, CXXC motif, transmembrane region, and ITAM. (C) Amino acid alignment of gCD3ε with Atlantic salmon (NM _001123622) and human (NM _000733.3) CD3ε sequences. The predicted signal peptide and domains are labeled. Residues similar/identical with gCD3ε are gray/black shade respectively. Ig-fold cysteine, CXXC motif and ITAM are boxed, and gaps (–) are indicated. Amino acid numbers are at right. (D) Comparison of ginbuna CD3ε with vertebrates CD3ε, CD3γ, CD3δ, and CD3ζ. Accession number of CD3 sequences are carp CD3ε (XM_019126514.1), takifugu (ta)CD3ε (NM_001037982.1), taCD3γ/δ (NM_001037983.1), taCD3ζ (XM_011608167.1), Japanese flounder (ja)CD3ε (XM_020094967.1), jaCD3γ (XM_020094974.1), jaCD3ζ (XM_020112573.1), salmon (sa)CD3ε (NP_001117094.1), saCD3δ (XM_014162423.1), saCD3ζ (XM_014164569.1), chicken CD3ε (NM_206904.1), mouse (mo)CD3ε (NM_007648.4), moCD3γ (NM_009850.2), moCD3δ, moCD3ζ (NM_001113391.2), human (hu)CD3ε (NP_000724.1), huCD3γ (EF444965.1), huCD3δ (EF444964.1), and huCD3ζ (AK128376.1). [file data_sheet_1.DOCX]

Supplementary Material

**Peculiar expression of CD3-epsilon in kidney of ginbuna crucian carp**

**Ryuichiro Miyazawa, Norifumi Murata, Yuta Matsuura, Yasuhiro Shibasaki, Takeshi Yabu, Teruyuki Nakanishi**

**Correspondence:**

Teruyuki Nakanishi, PhD

[nakanishi.teruyuki@nihon-u.ac.jp](file:///E:\data%20files\②論文関係\鱗移植\nakanishi.teruyuki@nihon-u.ac.jp)

# Supplementary Data

**Identification and characterization of ginbuna CD3ε**

To obtain the partial ginbuna CD3ε nucleotide sequences, we performed RT–PCR using primers for ginbuna *cd3e* (Table 1) designed using nucleotide sequence of zebrafish *cd3e* (NM_001326401) and *Paralichthys olivaceus* *cd3e* (AB081751). PCR was carried out in 40-µl reaction mixtures containing Prime STAR HS, with reaction conditions consisting of denaturation at 96°C for 2 min and 30 cycles of denaturation at 94°C for 10 s, annealing at 60°C for 10 s, polymerization at 72°C for 30 s, and extension at 72°C for 2 min. The PCR products were subcloned into the pGEM-T Easy plasmid vector by using a TA-Cloning method (Promega, Madison, WI, USA). After confirming through sequencing, 5’-RACE and 3’-RACE protocols (TaKaRa Bio, Shiga, Japan) were used to obtain full-length gCD3ε sequences using the primers shown in Table1. Nucleotide and amino acid sequences were analyzed using GENETYX-WIN version 9.0 and CLUSTALW. Similarity searches were performed using BLAST against the NCBI nonredundant protein database and the Protein Data Bank (PDB). Ig domains, CXXC motif and ITAM (Immunoreceptor Tyrosine-based Activation Motifs) were predicted using Simple Modular Architecture Research Tool (SMART). Phylogenetic analysis was performed using Molecular Evolutionary Genetics Analysis (MEGA).

**Immunoprecipitation and protein sequencing of ginbuna CD3**ε **by LC-MS/MS**

Immunoprecipitation was performed with a slight modification of previously described method (Yabu *et al*. 2015). Briefly, leukocytes from thymus and spleen were lysed by passing through 27-gauge needle and a syringe several times and then incubate for 1 h at 4 °C in 1 ml of 20 mM Tris–HCl (pH 7.5) containing 1% Nonidet P-40, 1% Triton X-100, 150 mM NaCl, 5 mM MgCl_2_, 1 mM EDTA, 1 mM EGTA, and 1 × proteinase inhibitor cocktail. Cellular debris was pelleted by centrifugation at 13 000 ×g for 15 min at 4 °C. The resulting cell lysates were first incubated with anti-gCD3ε, followed by incubation with protein G Sepharose (GE Healthcare, Piscataway, NJ, USA), sedimentation, and washing. The immunoprecipitant was resolved on SDS-polyacrylamide gel and visualized by silver staining (Wako chemicals, Osaka, Japan). A band excised from the gel was sent for sequence analysis by LC-MS/MS to Relyon Ltd. (Tokyo, Japan).

**Western blot analysis**

Nuclear fractions were extracted using a Nuclear Extract Kit (Active Motif, Carlsbad, CA, USA) according to the manufacturer’s instructions. The immunoprecipitated proteins were resolved on a SDS-polyacrylamide gel and electroblotted onto a PVDF membrane as described by Yabu *et al.* (2011). The membrane was incubated with 1:300 Anti-gCD3ε Ab 4°C for 10 hours, washed five times and stained with 1:2000 diluted HRP conjugated goat anti-rabbit IgG (Sigma-Aldrich, St. Louis, MO, USA) for 90 minutes at room temperature. The membrane was then washed five times and signals were detected using an ECL™ Western blotting detection kit (GE Healthcare, Piscataway, NJ, USA), according to the manufacturer’s protocol.

**Immunoabsorption test**

Specificity of the Anti-gCD3ε was confirmed by antigen absorption. 2 μg of the antibody was incubated with or without 20 μg of CD3e–ΔTM protein in 1 ml of PBS overnight at 4°C. Western blot was subsequently performed with the absorbed and unabsorbed antibody solution.

**RT-PCR analysis**

Total RNA was prepared from PBL, thymus, head-kidney, trunk-kidney, spleen, liver, ovary, intestine, skin and gill tissues using the ReliaPrep RNA Tissue Miniprep System (Promega Corporation, Madison, WI, USA) according to the manufacturer’s protocols and guidelines. cDNA was synthesized from total RNA from each sample using a High Capacity cDNA Reverse Transcription Kit (Applied Biosystems, CA, USA) according to the manufacturer’s protocols and guidelines. The PCR conditions were as follows: one cycle of 94 ^o^C for 2 min, 22-32 cycles at 94 ^o^C for 10 s, 55 ^o^C for 10 s and 72 ^o^C for 30 s. The PCR reactions were carried out in 20 ml mixtures containing 10 ml GoTaq® Green Master Mix (Promega Corporation, Madison, WI, USA), 200 nM of each primer, and 1 ml of cDNA. The PCR products were electrophoresed in 2% agarose gels and visualized by staining with 1 ng/ml ethidium bromide (Wako chemicals, Osaka, Japan). Images of the PCR products were photographed using AE-6932GXCF Printgraph (ATTO, Tkyo, Japan).

**Flow Cytometry**

5 × 10^6^ cells/ml of leukocytes from the various tissues were fixed with 2% paraformaldehyde (PFA) followed by cell membrane permeabilization with 0.1% saponin for 10 min. Cells were then incubated with 1:300 anti-gCD3ε antibody or Rabbit (DA1E) mAb IgG Isotype control (CST, MA, USA) for 45minat 4°C, washed three times and stained with 1:500 diluted Alexa Fluor^®^ 647 conjugated secondary antibody (Thermo Fisher Scientific Inc., MA, USA) against anti-gCD3ε antibody. The cells were then washed three times and served for flow cytometric analysis. [Lymphocytes](http://www.sciencedirect.com/topics/immunology-and-microbiology/lymphocytes) were gated on FS & SS dot plot and then analyzed using a FACS Canto flow cytometer (Becton Dickinson, NJ, USA).

Miyazawa *et al*. (2016) reported that anti-ginbuna CD4-1(6D1, rat) and CD8α (2C3, rat) mAbs cross react with carp and goldfish lymphocytes. For two-color immunofluorescence analysis of cell surface antigens along with CD3ε, kidney leukocytes were first incubated with mAbs against CD4-1(6D1, rat) and CD8α (2C3, rat) markers then fixed with 2% paraformaldehyde (PFA) followed by cell membrane permeabilization with 0.1% saponin for 10 min. Cells were then incubated with 1:300 anti-gCD3ε antibody for 45 min at 4°C, washed three times and stained with 1:500 diluted Alexa Fluor^®^ 488 donkey Anti-Rat IgG (H+L) antibody, [Alexa Fluor](http://www.sciencedirect.com/topics/biochemistry-genetics-and-molecular-biology/alexa-fluor)^®^ 488 goat Anti-mouse IgG (H+L) antibody and Alexa Fluor® 647 goat Anti-rabbit IgG (H+L) antibody (Thermo Fisher Scientific Inc., MA, USA) A donkey anti-rat IgG antibody was used for mAbs 2C3 and 6D1 along with a donkey anti-rabbit IgG antibody was used for anti-gCD3ε antibody. The cells were then washed three times. Lymphocytes were gated on FS & SS dot plot and then analyzed for double staining with the mAbs.

**Transcriptional Analysis of FACS Sorted Populations**

Leukocytes from spleen were labeled with anti-gCD3ε antibody as described above. Lymphocyte fraction of spleen leukocytes was gated and doublets discrimination was performed as described above. CD3ε^+^ cells were isolated by FACS Aria II cell sorter (Becton Dickinson, NJ, USA). Flow cytometry analysis of FACS sorted CD4-1 and CD8α exhibited high homogeneity and purity with more than 95%. Total RNA was extracted from 1× 10^6^ cells of FACS sorted cells using RNeasy® FFPE (Qiagen Hilden, Germany) according to the manufacturer’s protocols and guidelines. cDNA was synthesized from total RNA from each sample using a High Capacity cDNA Reverse Transcription Kit (Applied Biosystems, CA, USA) according to the manufacturer’s protocols and guidelines. mRNA expression analysis was performed as described above.

**Allo-antigen stimulation**

Allo-antigen stimulation was performed according to the method described by Toda et al. (26). After the stimulation, kidney leukocytes were incubated with anti-gCD3ε antibody followed by the second antibody as described above and used for FACS analysis.

***Edwardsiella tarda* infection**

100 μl of 1.2 x 10^7^ CFU/ml *Edwardsiella tarda* was i.p. injected into ginbuna. Three days after the injection, kidney leukocytes were incubated with anti-gCD3ε antibody followed by the second antibody as described above and used for FACS analysis.

**REFERENCES**:

Miyazawa R, Matsura Y, Shibasaki Y, Imamura S, Nakanishi T. Cross-reactivity of monoclonal antibodies against CD4-1 and CD8α of ginbuna crucian carp with lymphocytes of zebrafish and other cyprinid species. Dev Comp Immunol (2016) 80:15–23. doi:10.1016/j.dci.2016.12.002

Yabu T, Toda H, Shibasaki Y, Araki K, Yamashita M, Anzai H, et al. Antiviral protection mechanisms mediated by ginbuna crucian carp interferon gamma isoforms 1 and 2 through two distinct interferon gamma receptors. J Biochem (2011) 150:635–48. doi:10.1093/jb/mvr108

Yabu T, Shiba H, Shibasaki Y, Nakanishi T, Immamura S, Touhata K & Yamashita M. Stress-induced ceramide generation and apoptosis via the phosphorylation and activation of nSMase1 by JNK signaling. Cell Death Differ (2015) 22:258–273. doi: 10.1038/cdd.2014.128.

Supplementary Figures and Tables

**Supplementary Figure 1A**


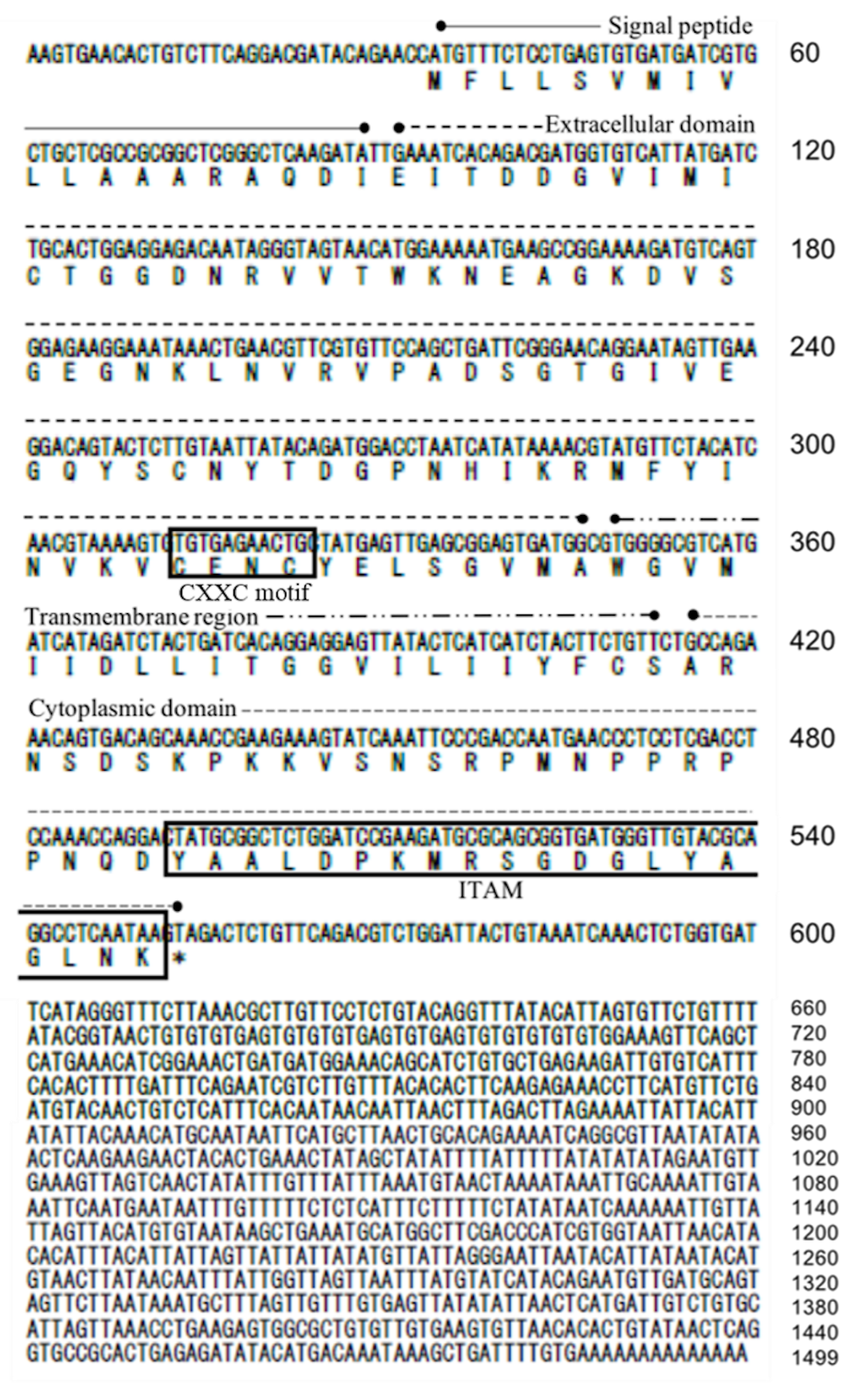


**Supplementary Figure 1B**


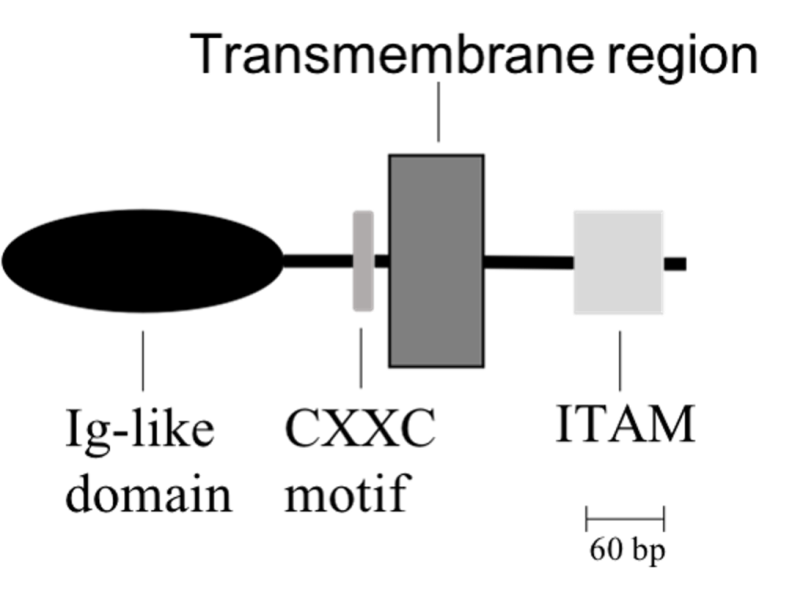


**Supplementary Figure 1C**


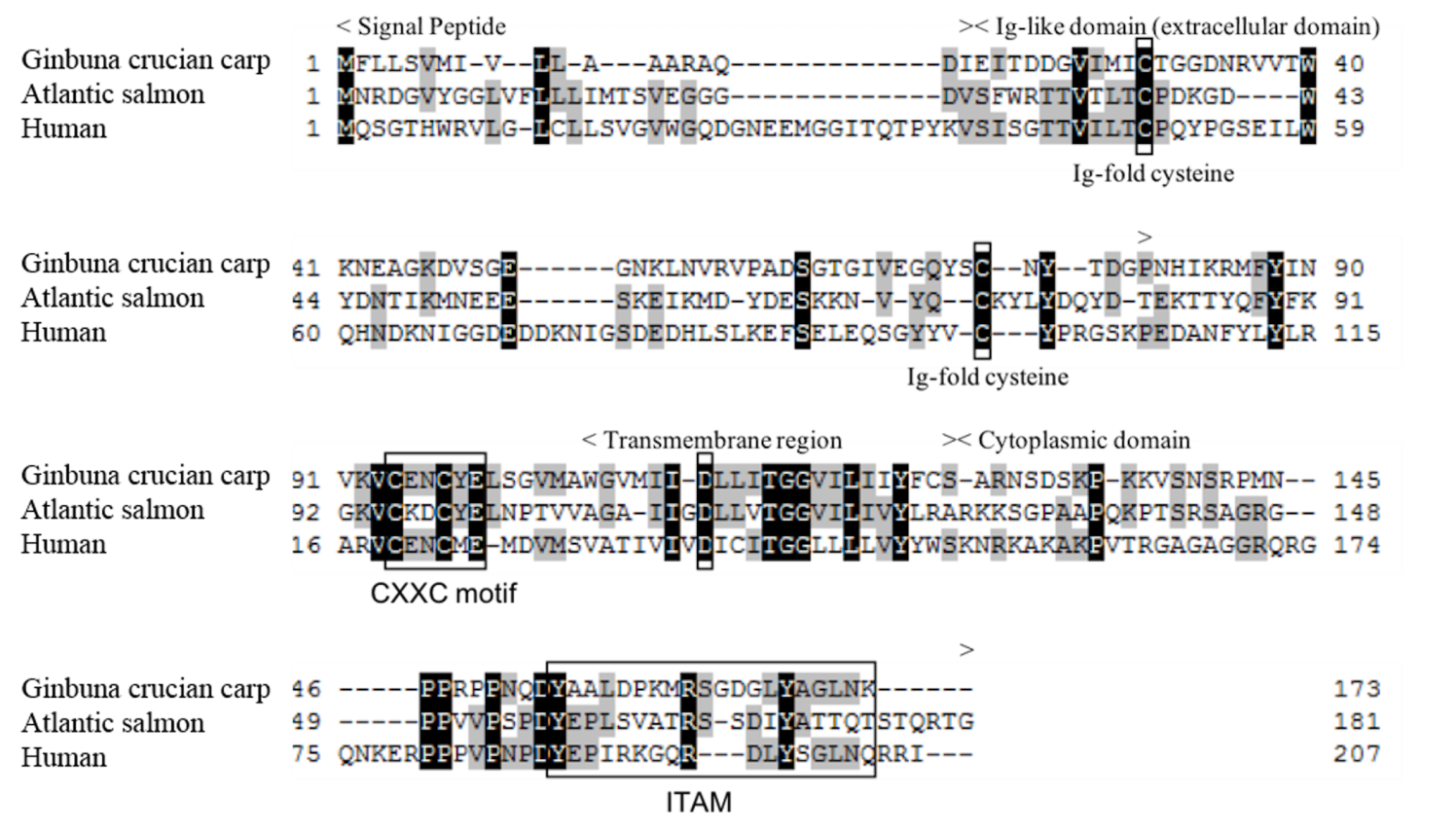


**Supplementary Figure 1D**


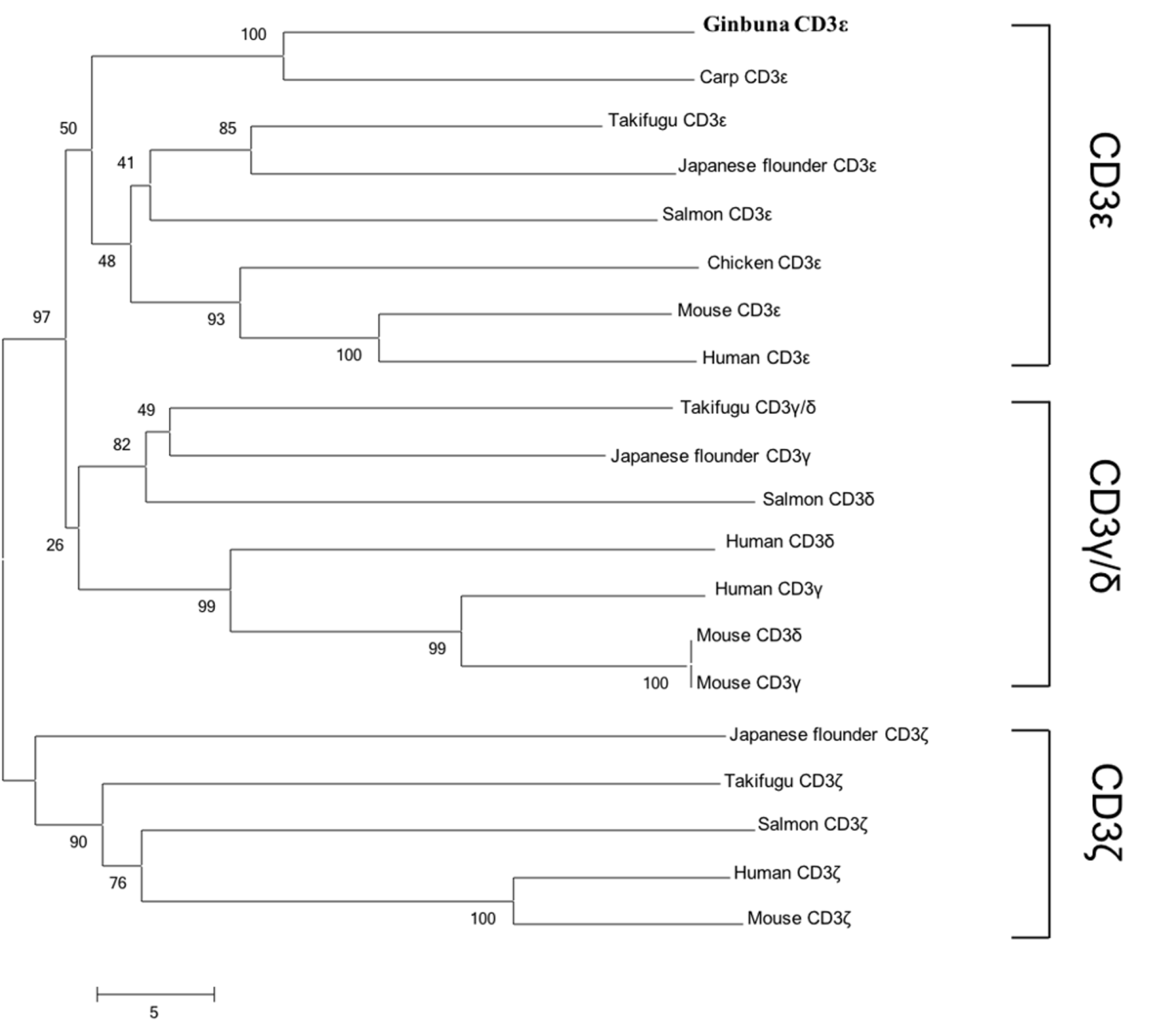


**Supplementary Figure 2**

**
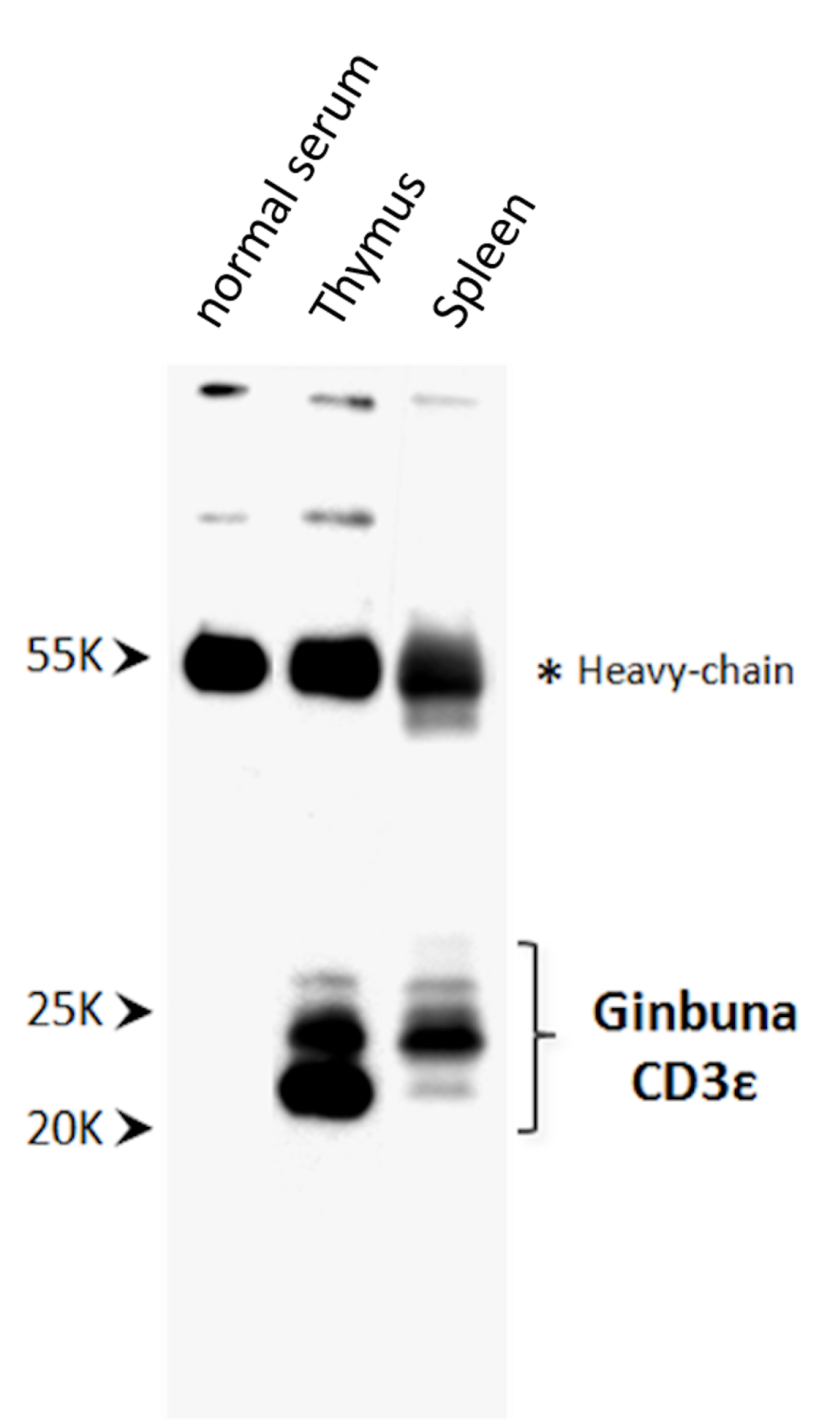
**

**Supplementary Figure 3**


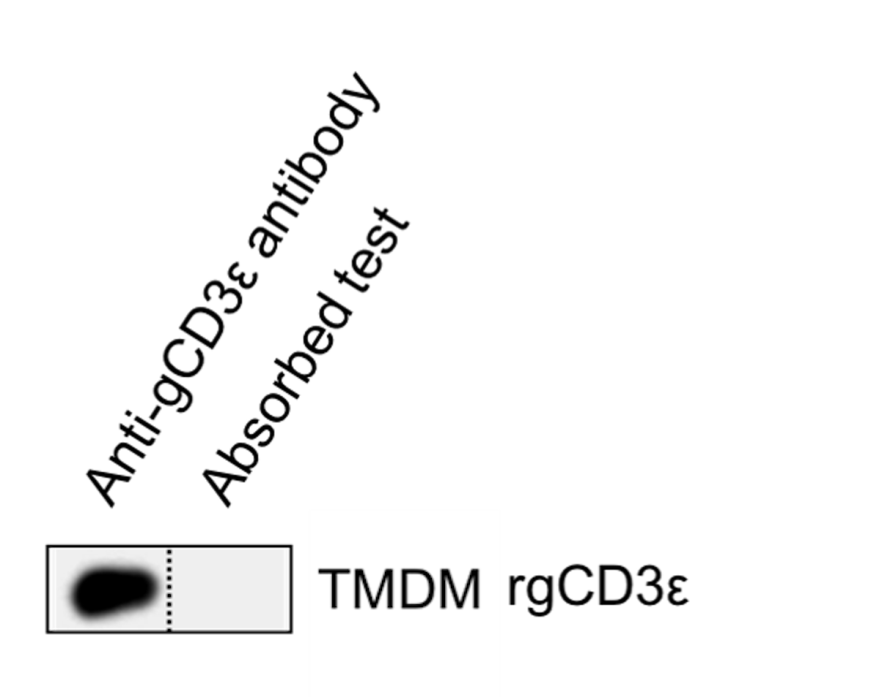


**Supplementary Figure 4A**


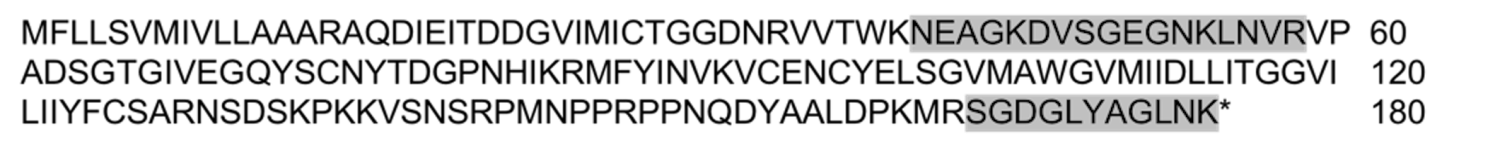


**Supplementary Figure 4B**

**
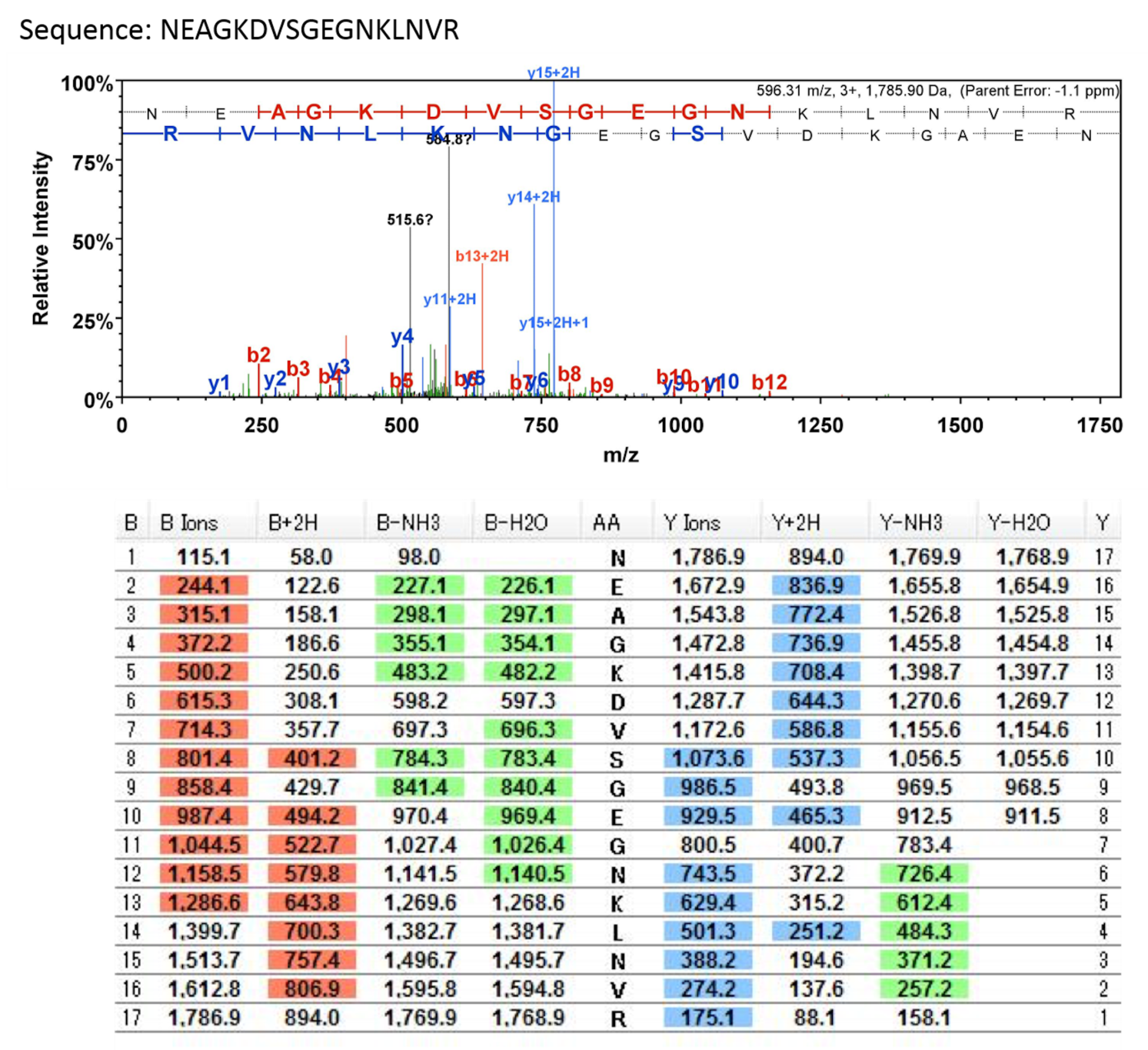
**

**Supplementary Figure 4C**

**
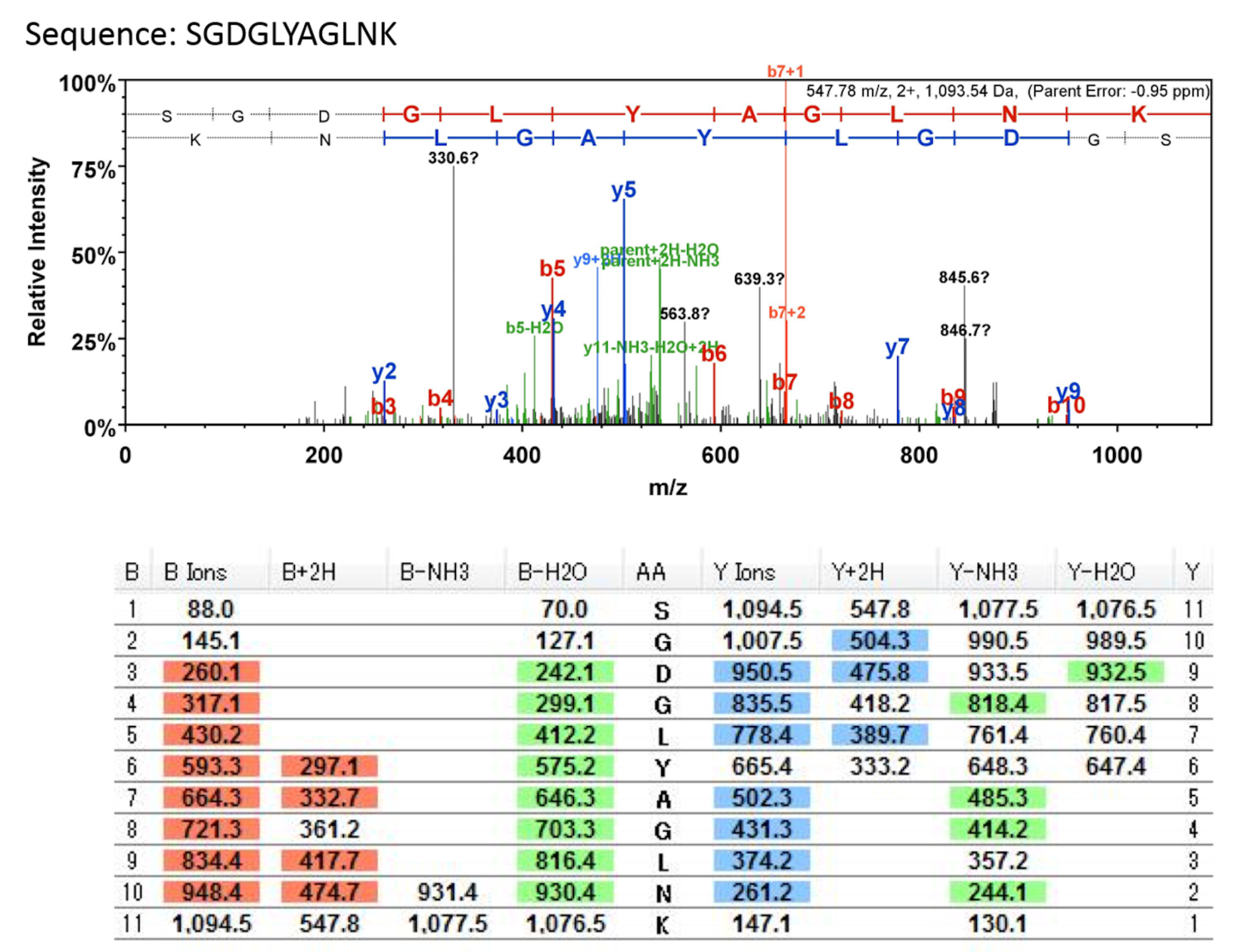
**

**Supplementary Figure 5**


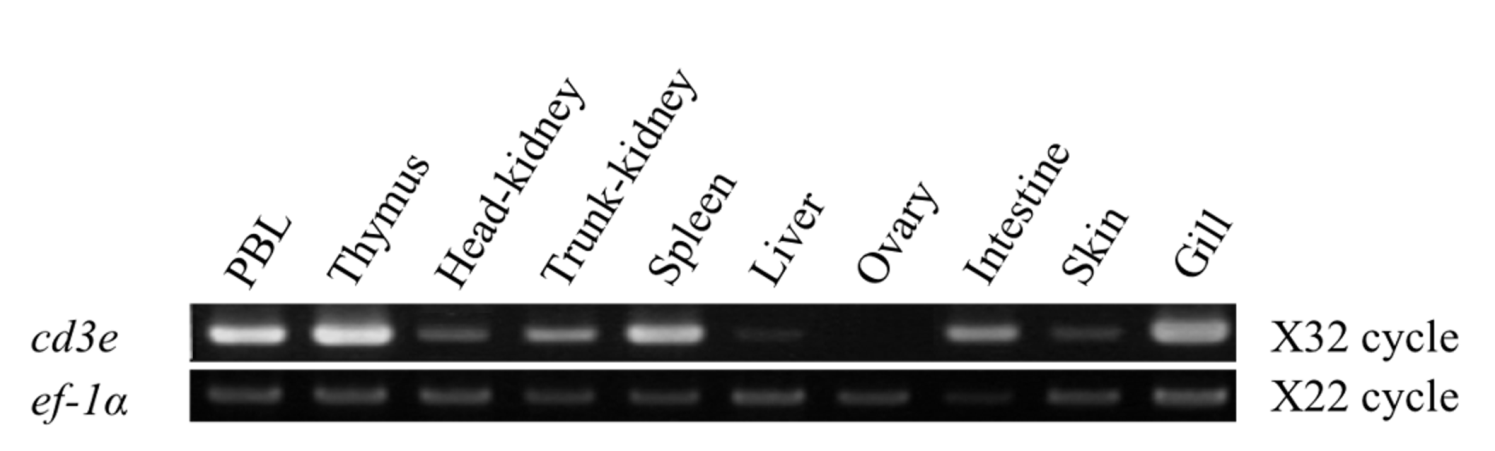


**Supplementary Figure 6**


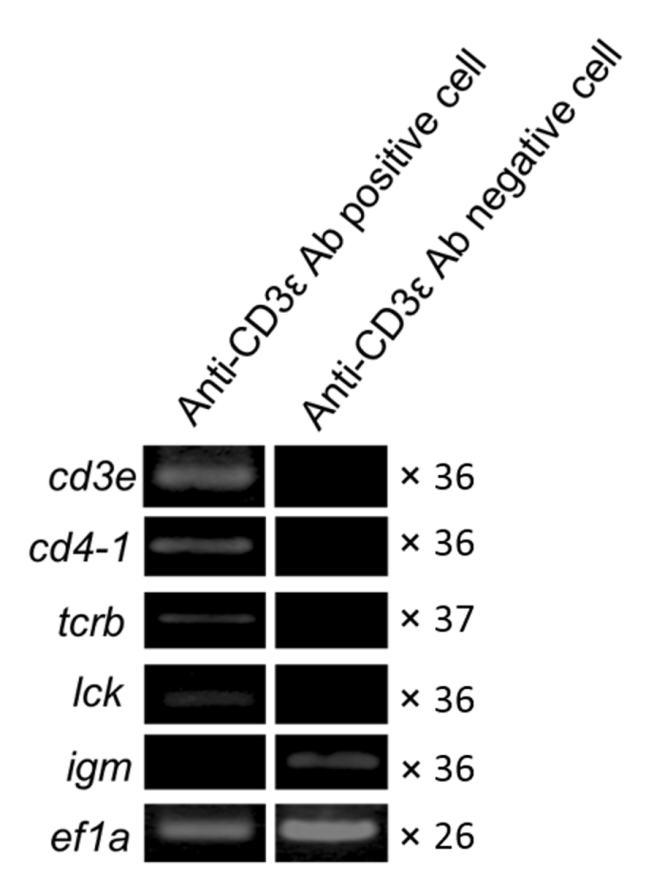


**Supplementary Figure 7A**


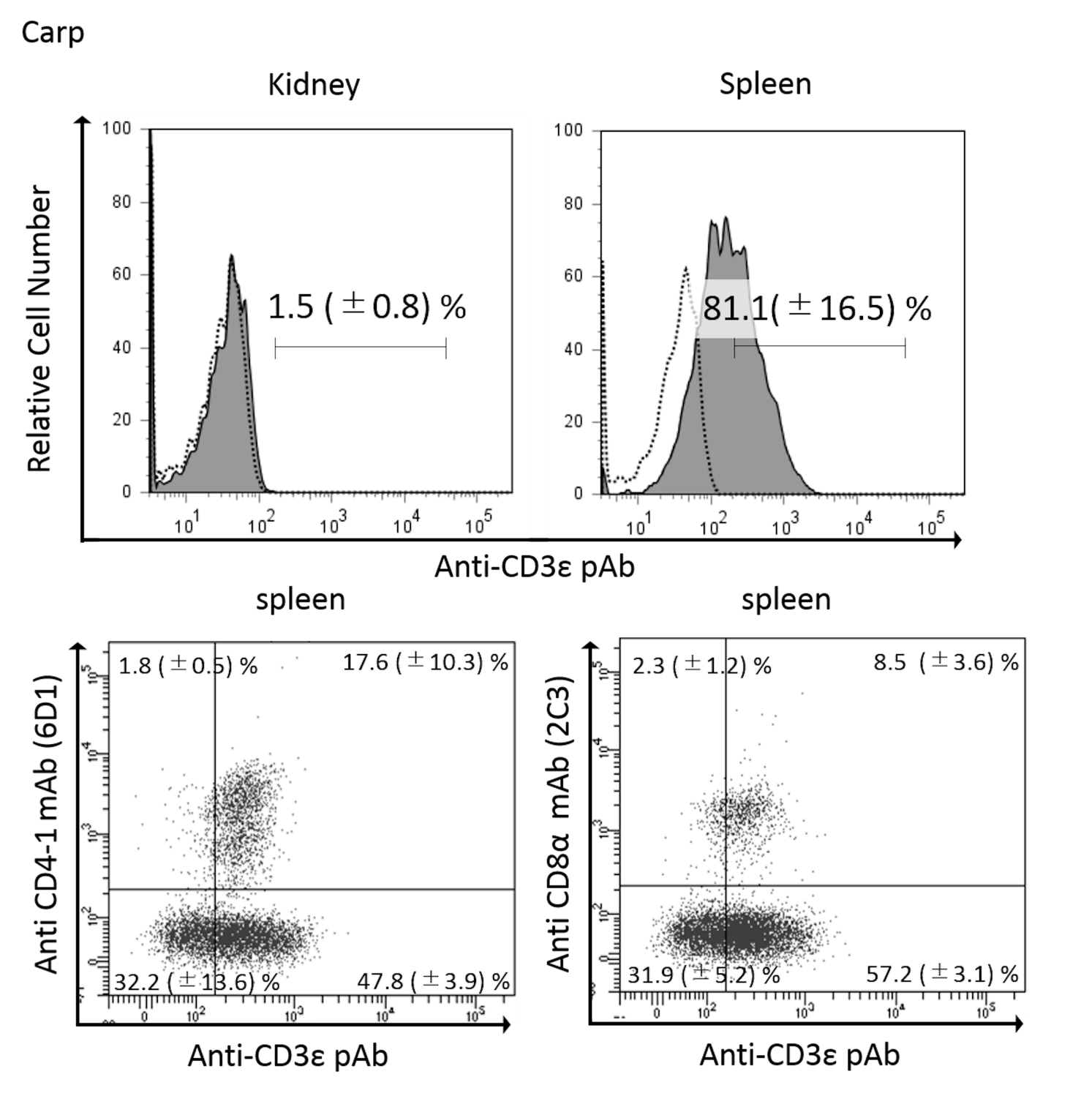


**Supplementary Figure 7B**


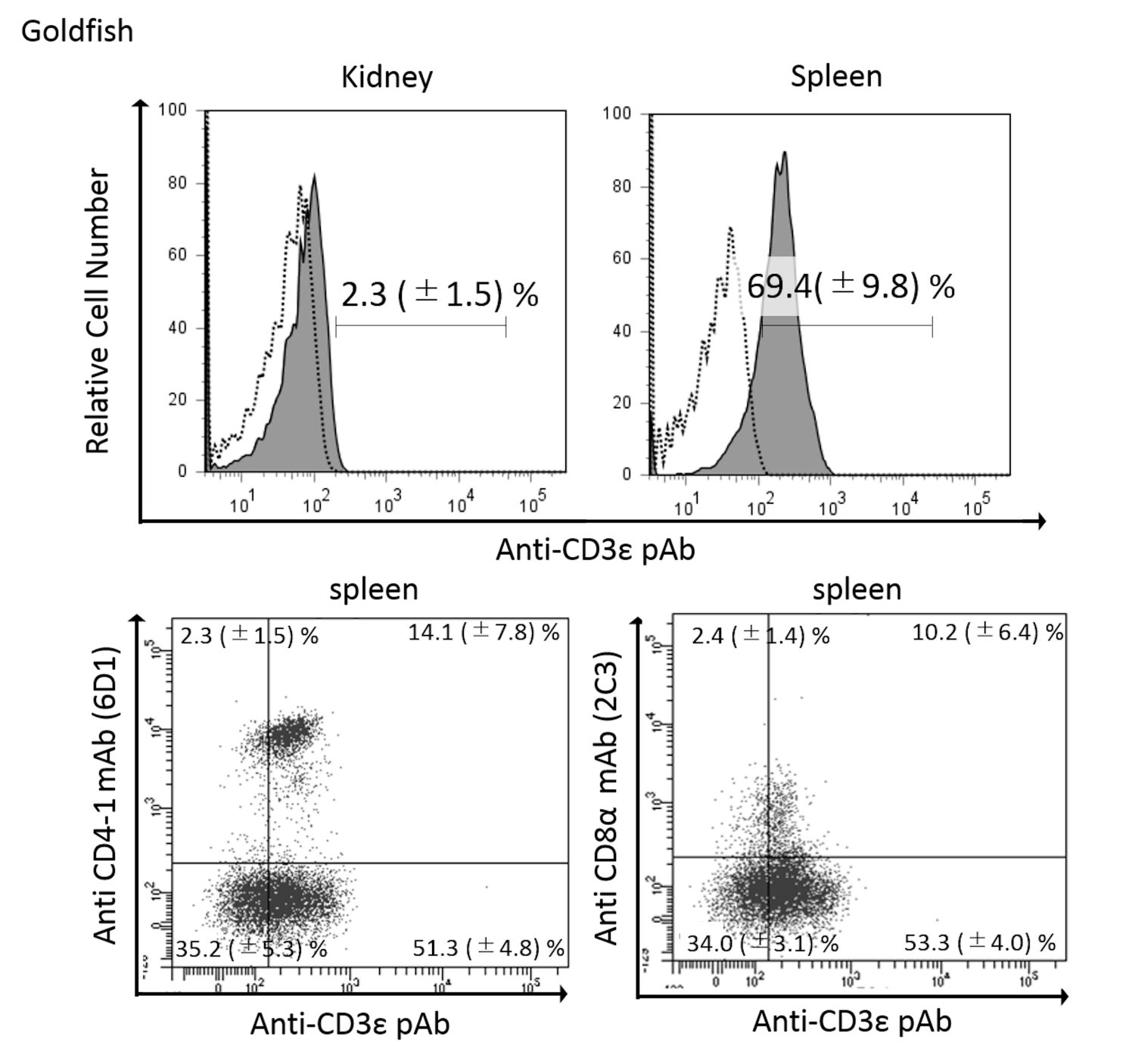


**Supplementary Figure 8**


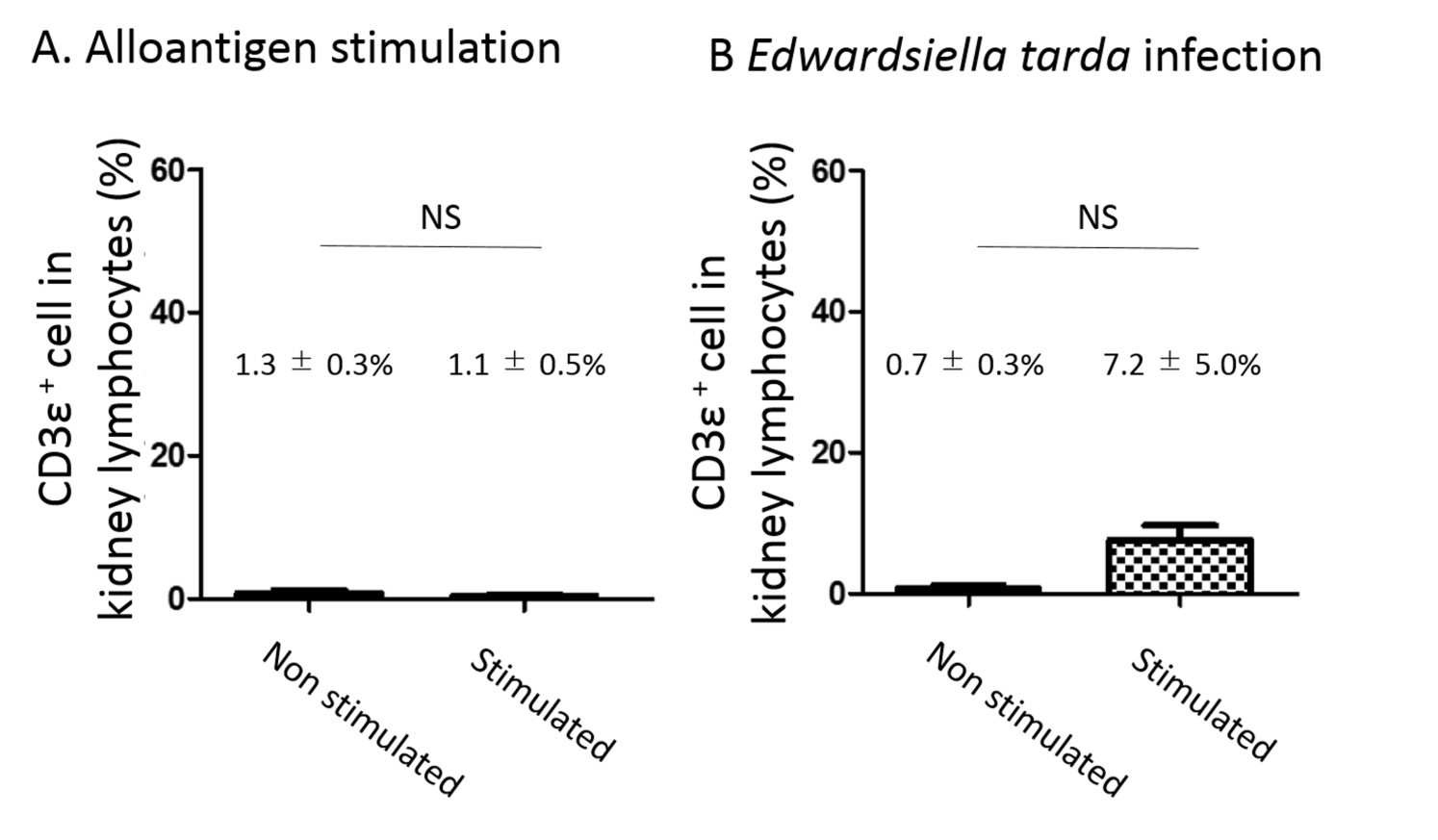


**Supplementary Figure 9A**


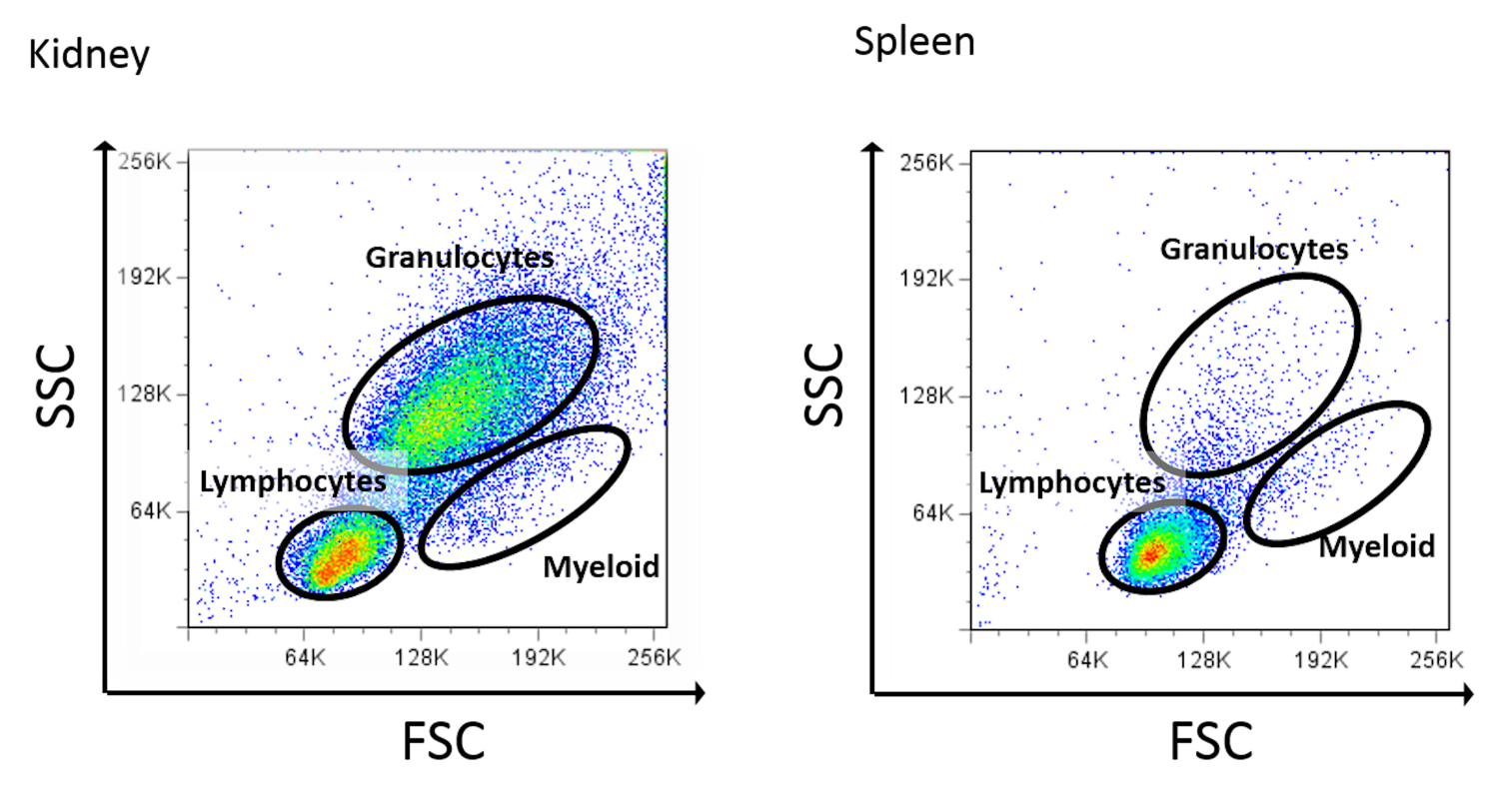


**Supplementary Figure 9B**


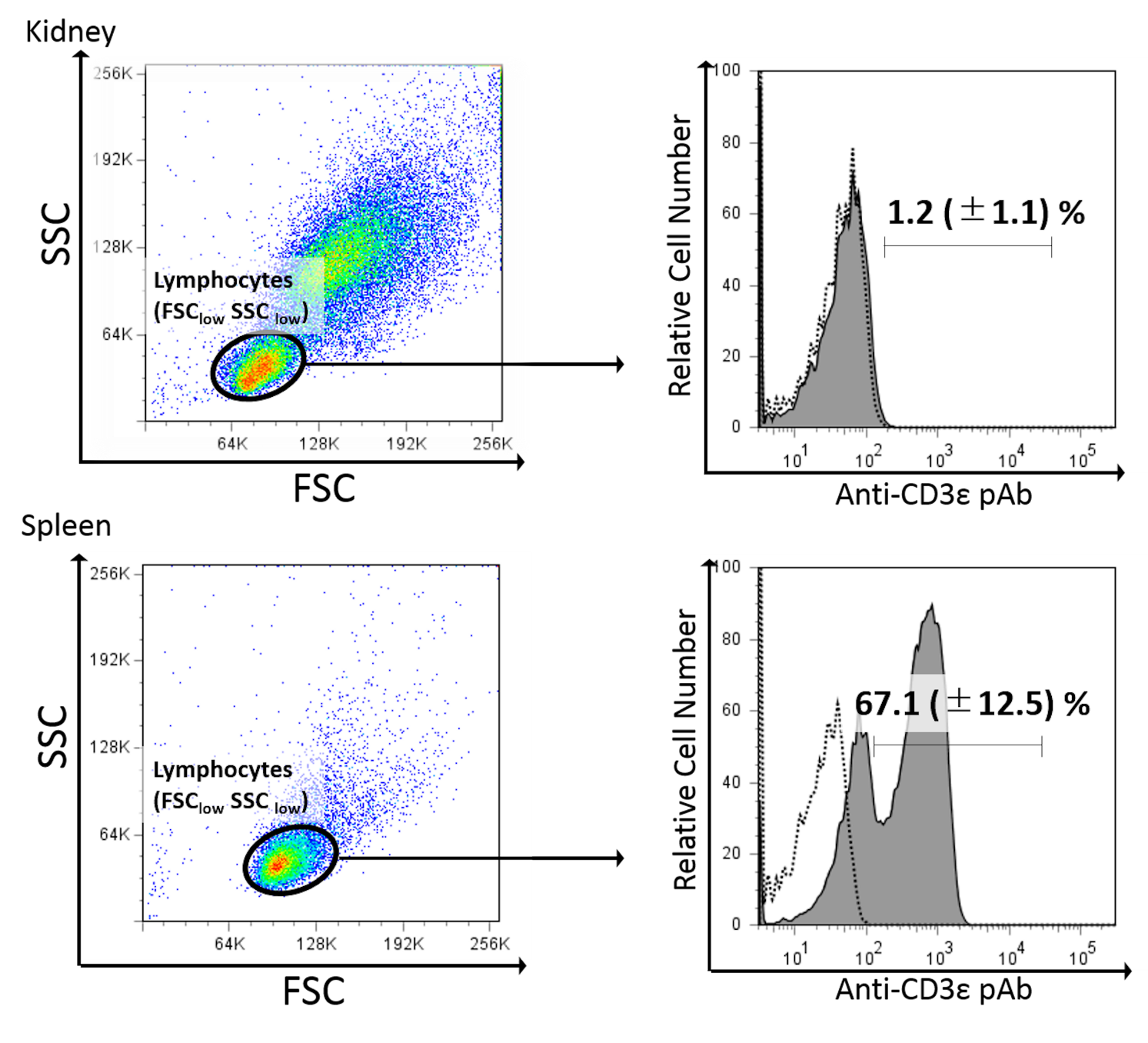


**Supplementary Figure 10**


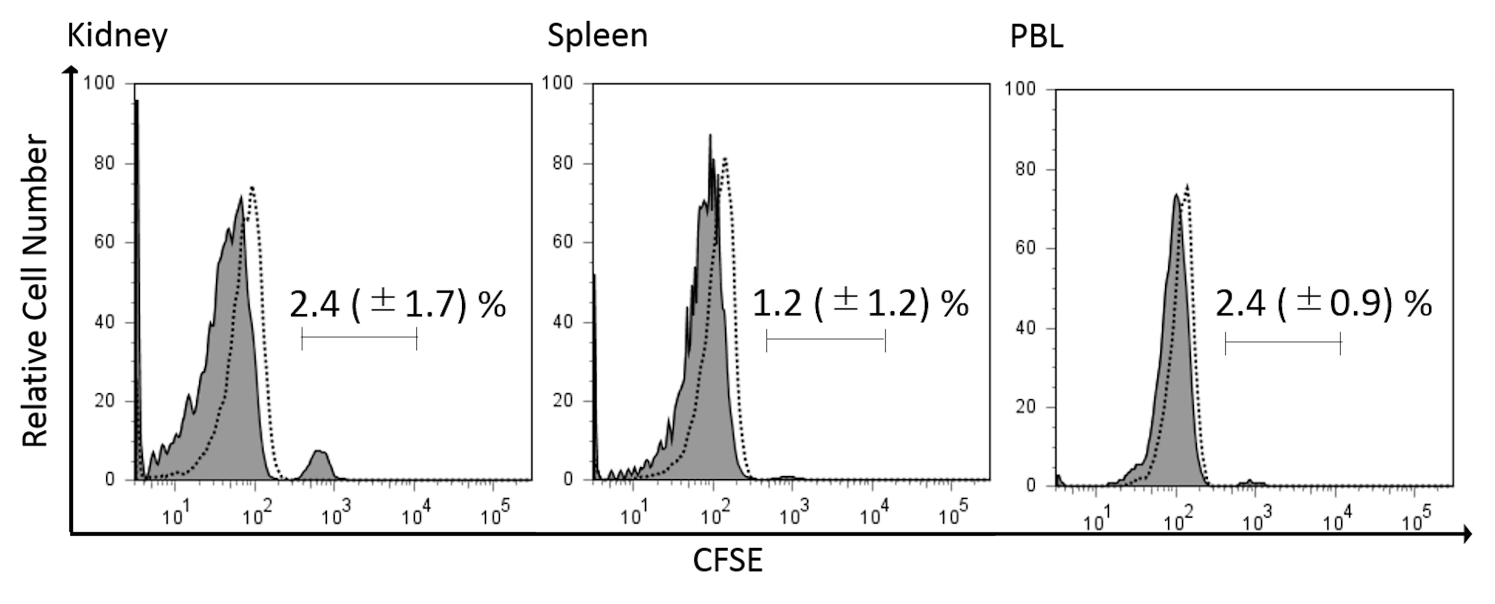


**Supplementary Figure 11**


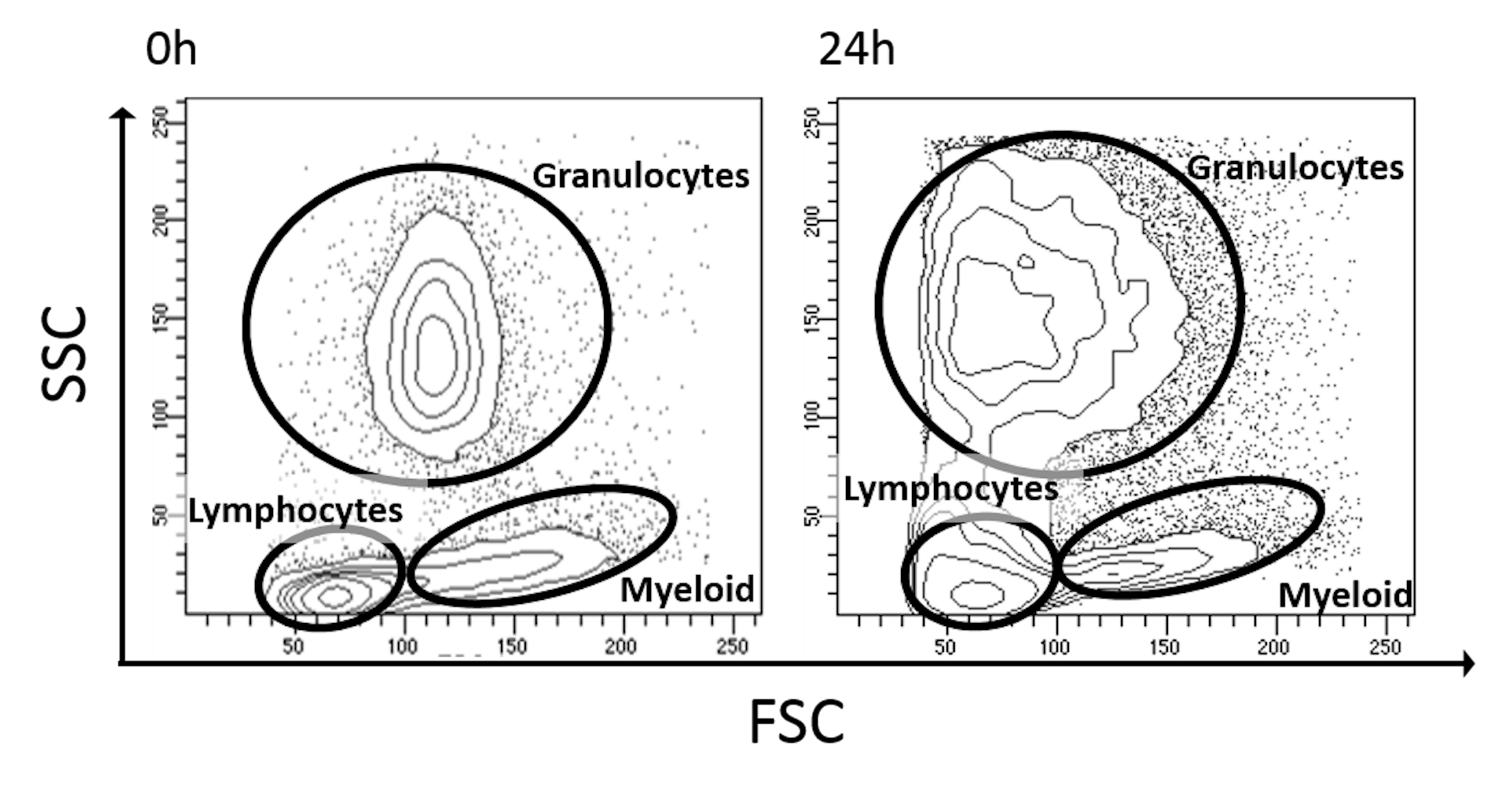


## Supplementary Figures

**
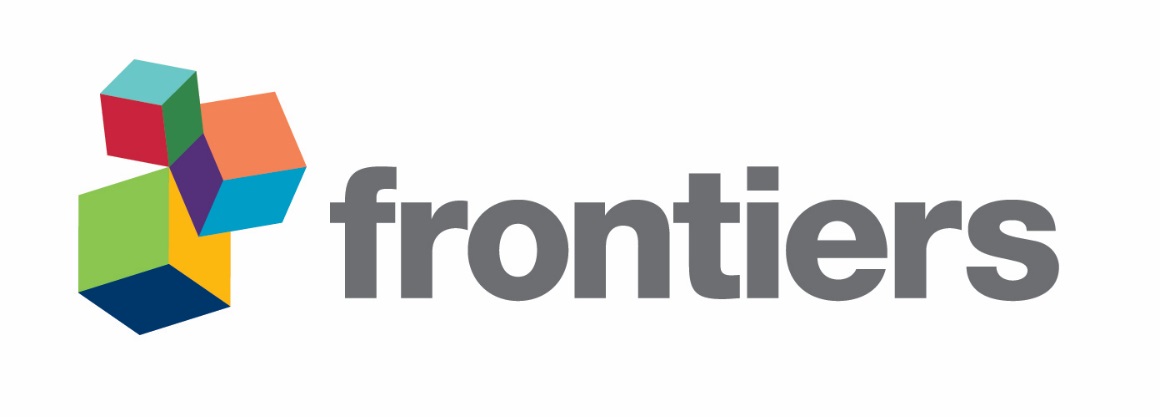
**

**Supplementary Figure 1** Ginbuna CD3ε sequence.

**(A)** Ginbuna CD3ε sequence. Nucleotide and amino acid sequence of ginbuna CD3ε are shown. Predicted signal peptide, Extracellular domain, Transmembrane region and Cytoplasmic domain are labeled, CXXC motif and ITAM are boxed. Amino acid numbers are at right.

**(B)** Schematic illustration of gCD3ε. Ginbuna CD3ε can be divided into Ig-like domain, CXXC motif, transmembrane region and ITAM.

**(C)** Amino acid alignment of gCD3ε with Atlantic salmon (NM _001123622) and human (NM _000733.3) CD3ε sequences. The predicted signal peptide and domains are labeled. Residues similar/identical with gCD3ε are gray/black shade respectively. Ig-fold cysteine, CXXC motif and ITAM are boxed, and gaps (-) are indicated. Amino acid numbers are at right.

**(D)** Comparison of ginbuna CD3ε with vertebrates CD3ε, CD3γ, CD3δ and CD3ζ. Accession number of CD3 sequences are: carp CD3ε (XM_019126514.1), takifugu (ta)CD3ε (NM_001037982.1), taCD3γ/δ (NM_001037983.1), taCD3ζ (XM_011608167.1), Japanese flounder (ja)CD3ε (XM_020094967.1), jaCD3γ (XM_020094974.1), jaCD3ζ (XM_020112573.1), salmon (sa)CD3ε (NP_001117094.1), saCD3δ (XM_014162423.1), saCD3ζ (XM_014164569.1), chicken CD3ε (NM_206904.1), mouse (mo)CD3ε (NM_007648.4), moCD3γ (NM_009850.2), moCD3δ, moCD3ζ (NM_001113391.2), human (hu)CD3ε (NP_000724.1), huCD3γ (EF444965.1), huCD3δ (EF444964.1) and huCD3ζ (AK128376.1).

**Supplementary Figure 2** Immuno-precipitation and Western Blot analysis. After immunoprecipitation of the thymus and spleen protein samples with ainti-gCD3ε Ab, the proteins were detected by western blotting with ainti-gCD3ε Ab. Both samples show some bands around 20-25 kDa expected to be gCD3ε and single band around 55 kDa expected to be heavy-chain.

**Supplementary Figure 3** Specificity test of rabbit serum by immune-absorption. Absorption test of gCD3ε Ab was performed using transmembrane deletion mutant (TMDM) recombinant gCD3ε protein. Western blot analysis shows no band when anti-gCD3ε Ab was absorbed with antigen (TMDM rgCD3ε, right), while the Ab not absorbed with the antigen shows positive band (left).

**Supplementary Figure 4** Protein sequencing by LC-MS/MS. Protein sequencing was determined using a protein band reactive with anti-gCD3ε Ab detected by Western blot. LC-MS/MS revealed 28 amino acid residues (gray highlight) and 16.1% of residues matched with gCD3ε amino acid sequence (A). Mass spectrum and fragmentation tables of each amino acid fragments are shown in (B) and (C), respectively. Peptide sequencing is indicated by matching b ion (red) and y ion (blue) fragments.

**Supplementary Figure 5** Expression analysis of CD3ε in ginbuna tissues by RT-PCR. Total RNA was prepared from PBL, thymus, head-kidney, trunk-kidney, spleen, liver, ovary, intestine, skin and gill tissues, and used for RT-PCR analysis. *ef-1a* was used as an internal control. Numbers to the right indicate PCR cycles.

**Supplementary Figure 6** Gene expression analysis of T and B cell related genes in sorted CD3ε^+^ lymphocytes. Spleen cells were stained with anti-CD3ε Ab as described. Lymphocytes fraction from spleen were gated on FS & SS dot plot and anti-CD3ε Ab positive cells were sorted by FACS. Total RNA was prepared from 1×10^6^ sorted cells and used for RT-PCR analysis. mRNA expression of *cd3e, cd4-, tcrb*, *lck, and igm* in sorted lymphocytes were shown. *ef-1a* was used as an internal control. Numbers to the right indicate PCR cycles.

**Supplementary Figure 7** CD3ε expression in tissues of other cyprinid species. Spleen and kidney leukocytes from carp (A) and goldfish (B) were stained with anti-CD4-1 and CD8α mAbs followed by Alexa Fluor® 488 anti-rat IgG, and stained with anti-gCD3ε Ab or anti-hZAP-70 mAb followed by 647 goat anti-rabbit IgG. Lymphocytes were gated on FS & SS dot plot. Mean ± SD of more than three independent experiments are shown.

**Supplementary Figure 8** Modulation of CD3ε expression. After allo-antigen stimulation (A) or *Edwardsiella tarda* infection (B), Kidney lymphocyte was stained with anti-gCD3ε as described above and analyzed by FACS. Mean ± SD of more than three independent experiments are shown. Statistical significance was calculated using t tests to each gene (ns, not significant p>0.05).

**Supplementary Figure 9** FS & SS dot plots of kidney and spleen leukocytes. Lymphocytes, myeloid cells and granulocytes were gated on FSC_low_ SSC_low_, FSC_high_ SSC_low_ and FSC _med_ SSC _high_ population, respectively (A). Lymphocytes from kidney and spleen were gated on FSC_low_ SSC_low_ population. The percentages of anti-CD3ε pAb positive cells were shown in the histogram (B).

**Supplementary Figure 10** Migration of donor cells in recipient organs. CFSE labeled donor cells were detected in recipient kidney, spleen and PBL on the histograms. Mean ± SD of more than three independent experiments are shown.

**Supplementary Figure 11** Effect of in vitro culture on the leukocytes composition. Before in vitro (0h) culture, leukocytes from kidney are composed of 46.2 % of granulocytes, 11.1 % of monocytes and 39.3 % of lymphocytes. Similarly, after in vitro (24h) culture, leukocytes from kidney are composed of with 50.0 % of granulocytes, 7.6 % of monocytes and 32.2 % of lymphocytes.

**Supplementary table for PCR primer**

| Expression analysis |  |  |  |
| --- | --- | --- | --- |
| *gef1α* F | ACCCCAAGGCTCTCAATCT | RT-PCR | AB491676 |
| *gef1α* R | TCAACGCTCTTGATGACACC | RT-PCR |  |
| *gcd3e* F | CTGCTATGAGTTGAGCGGAGTGAT | RT-PCR | LC378416 |
| *gcd3e* R | CTTCGGTTTGCTGTCACTGTTTCT | RT-PCR |  |
| *gtcrb* F | CCTGAAGCCCTCTGAAATCG | RT-PCR | AB186399 |
| *gtcrb* R | TGCTTCCAAGGCTCCATCTT | RT-PCR |  |
| *glck* F | CCATCCAGTCAAATACAGCAAA | RT-PCR | AB279594 |
| *glck* R | CTTTCTCAAACCCAAGGTCATC | RT-PCR |  |
| *gcd4-1* F | TACCGCGGGAACAACTTAAC | RT-PCR | AB331216.1 |
| *gcd4-1* R | TGTTGCTTCTGTTTGCTTCG | RT-PCR |  |
| *gigm heavy chain* F | TGGTTTCAGGATGGTAAGGA | RT-PCR | GU563726.1 |
| *gigm heavy chain* R | GGGTTCTTTACCATTGTCTCTG | RT-PCR |  |
